# Complexes of DOTAM with d^10^ Divalent Metal Ions: X‑ray Diffraction and NMR Studies in Solution and the Solid State

**DOI:** 10.1021/acs.inorgchem.6c01226

**Published:** 2026-05-28

**Authors:** Jakub Obuch, Ivana Císařová, Jiří Czernek, Jiří Brus, Petr Hermann

**Affiliations:** † Department of Inorganic Chemistry, Faculty of Science, Charles University, Hlavova 2030/8, 128 40 Prague 2, Czech Republic; ‡ Institute of Macromolecular Chemistry, Czech Academy of Science, Heyrovského náměstí 2, 162 00 Prague 6, Czech Republic

## Abstract

A detailed understanding of coordination asymmetry and metal–ligand bonding strength in macrocyclic complexes remains challenging, as subtle electronic differences often escape detection by conventional structural methods. Complexes of the tetrakis­(acetamide) cyclen derivative dotam with diamagnetic d^10^ metal ions Zn­(II), Cd­(II), and Hg­(II) were investigated in the solid state and in solution by single-crystal X-ray diffraction, multinuclear solid-state and solution NMR spectroscopy, and density functional theory calculations. The crystal structures reveal a progression from hexacoordinated Zn­(II), with only two coordinated pendant arms, to octacoordinated Cd­(II) and Hg­(II) complexes with distorted square-antiprismatic geometries. Solid-state ^13^C and ^15^N MAS NMR spectra agree well with the crystallographic models and GIPAW-DFT calculations. A key finding is the resolved splitting of amide carbonyl ^13^C resonances, attributed to residual ^13^C–^14^N dipolar coupling, which sensitively reports metal-induced perturbation of coordinated amide groups not evident from X-ray diffraction alone. Variable-temperature ^13^C NMR spectroscopy in aqueous solution revealed dynamic interconversion processes with activation parameters differing systematically from those of the corresponding DOTA complexes. Uniformly negative activation entropies support a common transition state consistent with transient octacoordination. Together, these results show that replacing acetate with acetamide pendant arms strongly affects both local coordination properties and solution dynamics.

## Graphical abstract

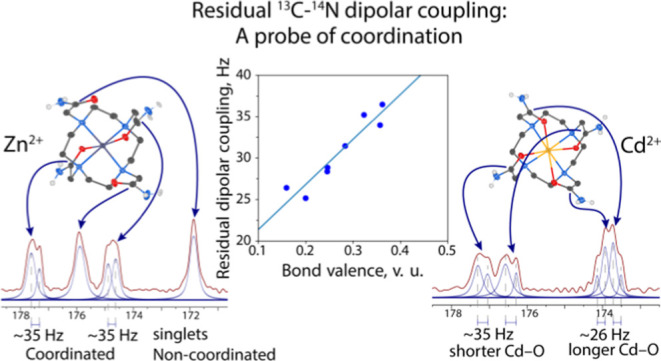

## Introduction

Macrocyclic ligands are widely used as a platform for the incorporation of heavy metal ions into various pharmaceuticals for use in vivo.^1–4^ One of the most employed parent macrocycles is 1,4,7,10-tetraazacyclododecane, also known as cyclen. Since most metal ions prefer higher coordination numbers (CNs), the nitrogen atoms of this macrocycle are often modified with pendant arms containing additional donor atoms. These coordinating pendant arms thus increase the stability and inertness of the resulting complexes, which ultimately enables the safe utilization of complexes of toxic heavy metal ions in vivo.^5^ These pendant arms can contain a variety of donor atoms allowing coordinating properties of the ligands to be fine-tuned to accommodate the appropriate metal ion. Moreover, the pendant arms offer ways to conjugate the complex to a biological vector for medicinal applications such as imaging and/or targeted therapy. The best-known macrocyclic ligand derived from cyclen is its tetraacetic acid derivative, DOTA. To obtain bifunctional ligands derived from DOTA, a modification of the methylene groups in either the macrocycle ring or a pendant arm is an obvious approach, but this is often synthetically difficult and produces a chiral compound. These complications are most commonly circumvented by employing an amide functional group instead of the carboxylate group. Consequently, biological vectors can be bound to the amide nitrogen atom directly or through a spacer.^6,7^

Another reason to employ an amide pendant arm instead of the carboxylate pendant arm is to modify the coordination properties of the ligand to optimize behavior of such a chelator toward certain metal ions. According to the Lewis acid–base theory, the uncharged amide group is softer than the negatively charged carboxylate. Thus, ligands containing amide groups are preferred for the complexation of softer metal ions, i.e., heavy metal ions such as Pb­(II),^8,9^ Hg­(II),^10,11^ or Bi­(III).^12,13^ The radioactive isotopes of these metal ions are employed in emerging radiopharmaceuticals for imaging and/or radiotherapeutical applications.^9,14,15^

The simplest cyclen derivative bearing four amide pendant arms, dotam, has already been studied from multiple points of view. Its Gd­(III) and Mn­(II) complexes were studied as potential MRI contrast agents,^16,17^ and the thermodynamic stability and kinetic inertness of these complexes were proved to be sufficient for in vivo applications.^18,19^ Exploiting the presence of amide protons, the Fe­(II), Co­(II), and Ni­(II) complexes of dotam were studied as model PARACEST MRI contrast agents.^20–23^ The solid-state structures of Zn­(II), Cd­(II), Hg­(II), and Pb­(II) complexes were determined and show variations in the coordination polyhedra, going from hexacoordinated octahedrons to octacoordinated twisted square antiprisms.^24,25^ We have recently studied the Hg­(II) complexes by multinuclear solid-state NMR spectroscopy and single-crystal X-ray diffraction, and we found that the coordination polyhedra in the Hg­(II) complexes are highly variable.^26^ We identified two distorted octacoordinated and one heptacoordinated Hg­(II) centers. However, the solution dynamics of complexes of dotam with divalent metal ions have been less investigated and no comparison has been done with related complexes; thus, no information on the mechanism of any isomer interconversion is available. Moreover, data for the Zn­(II) complex have been obtained in DMF instead of water,^24^ as the most important solvent. Thus, the variable-temperature (VT) NMR data were redetermined in water or water/methanol mixtures to allow better comparison.

Despite the existence of several single-crystal X-ray structures of dotam complexes with divalent metal ions, important aspects of their coordination behavior remain insufficiently understood. While crystallography provides a static description of atomic positions, it offers limited sensitivity to subtle differences in local electronic structure, weak metal–amide group interactions or coordination asymmetry that do not translate into clearly distinguishable structural parameters. Solution-state NMR spectroscopy, while sensitive to electronic environments, averages over rapid molecular motions and, therefore, renders nonequivalent coordination sites indistinguishable. This obscures asymmetric metal–ligand interactions and pendant-arm binding modes. As a result, key features of metal-dependent coordination behavior remain inaccessible to either method alone. Solid-state NMR (ss-NMR) spectroscopy overcomes these limitations by retaining site-specific sensitivity to local electronic environments in rigid lattices. By accessing NMR observables that are averaged out in solution yet not explicitly encoded in crystallographic models, the ss-NMR provides a crucial link between static structures and solution dynamics, enabling a more complete description of metal–ligand coordination in dotam complexes.

In the present work, we aim to better understand structures of dotam complexes with diamagnetic d^10^ transition metal ions, Zn­(II), Cd­(II), and Hg­(II), in both solution and the solid state. We employed multinuclear solution and ss-NMR spectroscopy complemented by quantum-chemical calculations. Furthermore, we explored the applicability of NMR crystallography for molecular complexes that are not routinely investigated by this technique. We compared properties of these complexes with data for complexes of DOTA with the same metal ions studied previously^27^ to better understand the effects caused by replacing the acetate with acetamide pendant arms.

## Results

### Synthesis

During attempts to isolate M­(II)-dotam complexes (*M* = Zn, Cd, and Hg) as crystalline chloride salts, tetrachlorometallate salts with [MCl_4_]^2–^ counteranions were repeatedly formed. This approach, however, afforded the target complexes only in low yields, and some free ligand was recovered from the mother solutions. The reactions were therefore repeated using a larger excess of the corresponding metal chlorides in order to drive the complexation to completion. Under these conditions, the title complexes were obtained in high yields. Single crystals suitable for X-ray diffraction analysis were prepared by slow cooling of hot aqueous solutions of the complexes. The same crystalline phases were also used for the solid-state NMR measurements (see below).

### Single-Crystal X-ray Diffraction

Two new compounds were prepared in a form suitable for X-ray structural analysis, [Zn­(dotam)]­[ZnCl_4_]·2.5H_2_O and [Cd­(dotam)]­[CdCl_4_]·0.5H_2_O. The experimental details for the diffraction analysis are provided in Table S1. Structures of two [Hg­(dotam)]^2+^ salts used here for comparison were described in our recent paper.^26^

The compound [Zn­(dotam)]­[ZnCl_4_]·2.5H_2_O crystallized in a monoclinic *P*2_1_/*c* space group. The asymmetric unit contains one [Zn­(dotam)]^2+^ cation (Figure 1), one [ZnCl_4_]^2–^ counteranion, and 2.5 water molecules of crystallization. The Zn­(II) ion in the [Zn­(dotam)]^2+^ cation is hexacoordinated by four nitrogen donor atoms (Zn–N bond lengths range from 2.226 to 2.267 Å) and two oxygen donor atoms (Zn–O bond lengths are 2.080 and 2.085 Å) originating from two “*trans*”-located acetamide pendant arms, forming a distorted octahedral environment. The Zn­(II) ion in the [ZnCl_4_]^2–^ counteranion is bound in an approximately tetrahedral fashion. More geometric parameters are given in Tables S2 and S3 and the crystal packing is shown in Figure S1.

**Figure 1:**
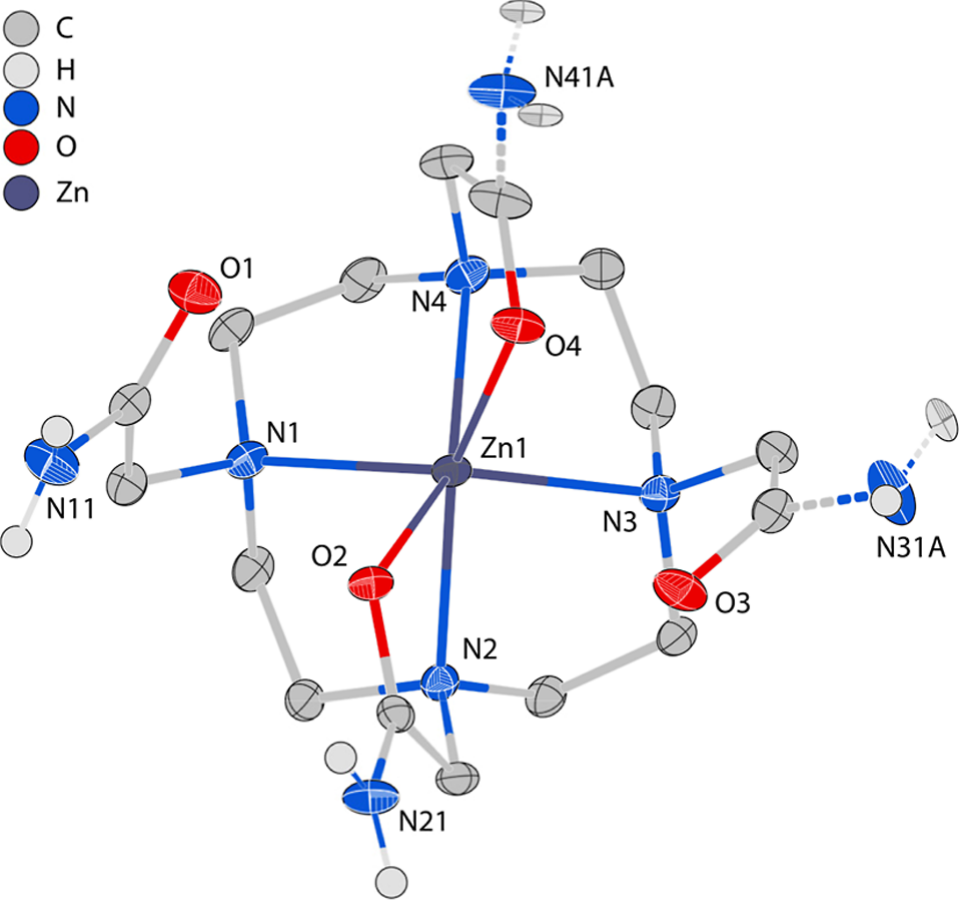
Structure of the [Zn­(dotam)]^2+^ cation in [Zn­(dotam)]­[ZnCl_4_]·2.5H_2_O in the solid state. Carbon-bound hydrogen atoms and minor parts of the disorders are omitted for clarity. Dashed bonds represent connectivity in the major part of the disordered amide nitrogen atoms. Thermal ellipsoids are drawn at the 50% probability level.

The compound [Cd­(dotam)]­[CdCl_4_]·0.5H_2_O crystallized in a monoclinic *P*21/*n* space group. The asymmetric unit contains one [Cd­(dotam)]^2+^ cation (Figure 2), one [CdCl_4_]^2–^ counteranion, and 0.5 water molecule of crystallization. The Cd­(II) ion in the [Cd­(dotam)]^2+^ cation is octacoordinated by four nitrogen donor atoms (Cd–N bond lengths range from 2.411 to 2.457 Å) and four acetamide oxygen donor atoms to form a twisted square antiprism (TSA). Two opposite Cd–O bonds (2.322 and 2.371 Å) are significantly shorter than the other two (2.584 and 2.500 Å), creating a [6 + 2] coordination fashion around the central metal ion. The Cd­(II) ion lies between the N_4_ and O_4_ planes, formed by the nitrogen and oxygen donor atoms, respectively, almost directly on the line connecting the centroids of these two planes closer to the nitrogen plane (Cd–N_4_ and Cd–O_4_ distances are 1.246 and 1.322 Å, respectively). The [CdCl_4_]^2–^ counteranion exhibits an approximately tetrahedral arrangement. More geometric parameters can be found in Tables S4 and S5, and the crystal packing is shown in Figure S3.

**Figure 2:**
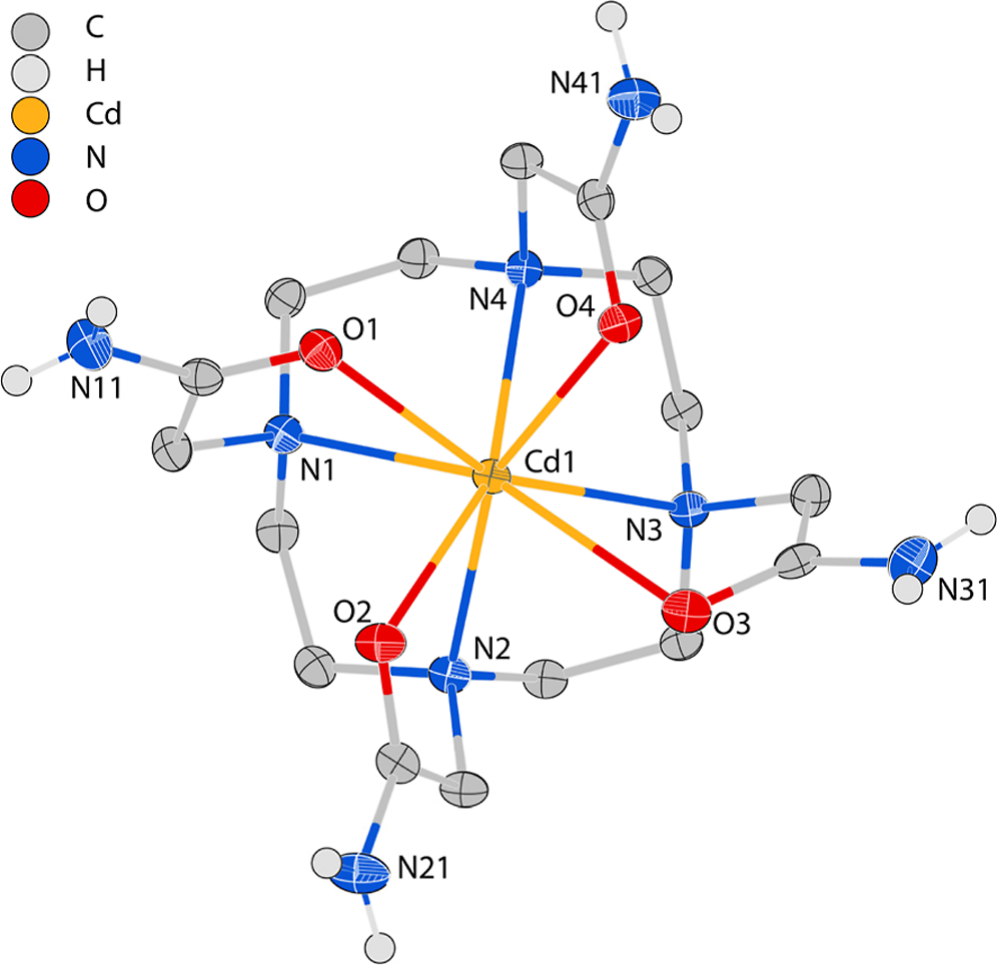
Structure of the [Cd­(dotam)]^2+^ cation in [Cd­(dotam)]­[CdCl_4_]·0.5H_2_O in the solid state. Carbon-bound hydrogen atoms are omitted for clarity. Thermal ellipsoids are drawn at the 50% probability level.

### NMR Crystallography

The ss-NMR data were recorded on the bulk samples from which the single crystals for X-ray diffraction were chosen. To ascertain the phase identity of the bulk sample, five to ten crystals from the same synthetic batch were measured on the diffractometer, all giving the same experimental crystal parameters. The ^13^C and ^15^N ss-NMR data are presented below. Unfortunately, extensive signal overlap in the ^1^H NMR spectra prevented any reliable signal assignment and meaningful interpretation (Figure S8).

The ^13^C CP/MAS NMR spectra (Figure 3) of all complexes are fully consistent with the crystallographic symmetry, displaying 16 resonances that account for the number of chemically distinct carbon sites in the asymmetric unit, including accidental overlap. This agreement confirms the correctness of the structural models and the phase purities of the bulk samples. The overall chemical shift patterns are well reproduced by GIPAW-DFT calculations, providing a solid basis for NMR crystallography analysis. Closer inspection of the carbonyl region reveals a systematic splitting of some ^13^C resonances into asymmetric doublets (see Figure S7). This behavior cannot be attributed to chemical nonequivalence, as the number and relative intensities of the signals remain consistent with crystallographic symmetry. Static structural disorder can also be excluded since the Cd­(II) complex shows no crystallographic disorder, yet all four carbonyl resonances are split. Furthermore, the persistence of the splitting over the accessible temperature range rules out dynamic exchange processes on the NMR time scale.

**Figure 3:**
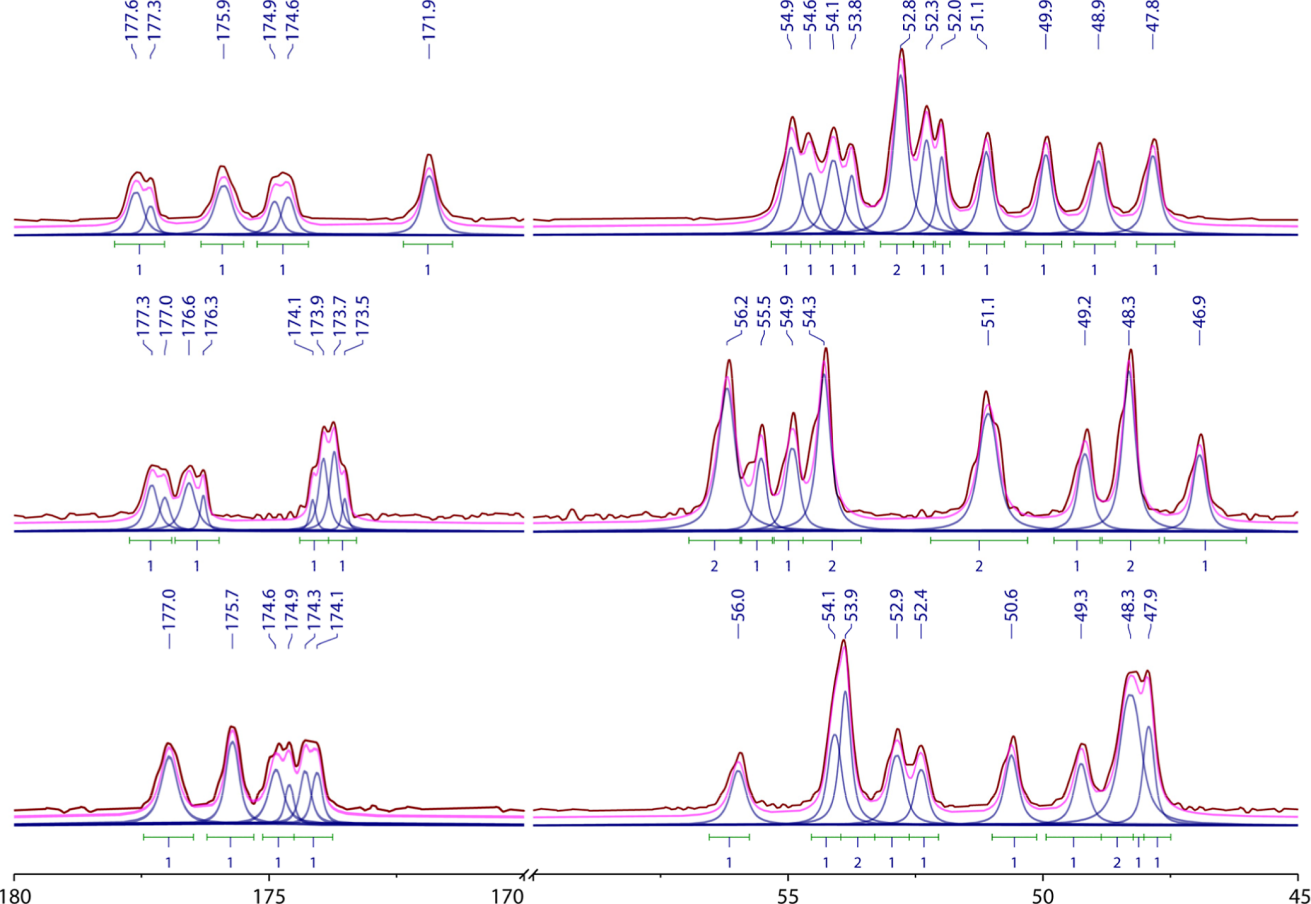
^13^C CP/MAS NMR spectra (126 MHz, 10 kHz) of [Zn­(dotam)]­[ZnCl_4_]·2.5H_2_O (top), [Cd­(dotam)]­[CdCl_4_]·0.5H_2_O (middle), and [Hg­(dotam)]­[Hg_3_Cl_8_]·3H_2_O (bottom). Color code: maroon: experimental spectra, blue: deconvoluted resonances of individual sites, magenta: sum of the deconvoluted resonances.

The observed splitting of some carbonyl ^13^C resonances in dotam complexes can be consistently interpreted in terms of residual ^13^C–^14^N dipolar coupling,^28^ whose manifestation is strongly dependent on the quadrupolar interaction and relaxation behavior of the directly bonded amide ^14^N nuclei. Internuclear dipolar coupling is normally averaged out by MAS. However, the molecule-fixed quadrupolar interaction is not negligible compared to the lab-frame Zeeman interaction and, therefore, the dipolar coupling appears in the spectrum, as the nucleus is not quantized exactly along the static magnetic field. Sample spinning thus cannot remove dipolar coupling if the quantization direction depends on molecular orientation. Moreover, while the magnitude of the ^13^C–^14^N coupling is governed primarily by the local electronic structure of the C–N bond, the extent to which this coupling appears as a resolved multiplet in the ^13^C spectrum is controlled by site-specific quadrupolar relaxation of the ^14^N nucleus. Consequently, the observed variability of the splitting across different C–N pairs reflects a combination of genuine differences in dipolar coupling constants and differences in the quadrupolar coupling constants arising from nonequivalent local environments of the amide nitrogen atoms.

Importantly, the appearance of the ^13^C–^14^N splitting correlates systematically with metal coordination in the dotam framework. In the Zn­(II) complex, only carbonyl groups directly involved in metal coordination exhibit detectable splitting, whereas in the Cd­(II) complex all four coordinated carbonyl groups give rise to resolved doublets. This behavior indicates that metal coordination modifies the electric field gradient at the amide nitrogen, most likely through changes in C–N bond polarization, planarity of the amide group, and local symmetry. These effects can be seen most prominently in the O–C–N angles of amide functional groups. In more strongly coordinated pendants, this angle is close to ∼121°, whereas in less strongly coordinated pendants this angle grows to ∼124°. These effects reduce the efficiency of quadrupolar relaxation, allowing the underlying residual dipolar coupling to be observed. Furthermore, carbonyl groups associated with longer metal–oxygen distances display smaller apparent coupling constants, suggesting that the ^13^C–^14^N interaction is sensitive to subtle variations in metal–ligand geometry and bonding strength. This assignment is further supported by the ^13^C CP/MAS NMR spectrum of the free dotam ligand, in which the carbonyl resonance at ca. 178 ppm is essentially symmetric and exhibits only slight broadening attributable to the overlap of two nonequivalent carbonyl groups present in the crystallographic unit cell (Figure S9).^29,30^ Thus, the pronounced splitting observed for the metal complexes is not an intrinsic feature of the free ligand but arises upon metal-induced perturbation of the coordinated amide groups.

The absence of analogous splitting in related DOTA complexes,^27^ which lack directly bonded amide nitrogen atoms, provides strong support for this assignment and rules out alternative explanations based on purely carbonyl-centered effects. Taken together, these observations demonstrate that the resolved ^13^C–^14^N splitting in dotam complexes serves as a sensitive probe of coordination of amide oxygen donor atoms, metal–ligand bonding asymmetry, and local lattice or metal-induced perturbation at the amide nitrogen atom, rather than reflecting a uniform intrinsic property of the ligand framework. This highlights the important role of amide nitrogen atoms in even *O*-coordinated amide groups and their quadrupolar characteristics in shaping the ss-NMR response of dotam-type chelators.

Beyond confirming the crystallographic models, the ^15^N CP/MAS NMR spectra provide a sensitive, site-specific probe of coordination asymmetry and metal–ligand bonding that is not directly accessible from diffraction data alone. While both the Zn­(II) and Cd­(II) complexes exhibit the expected number of amine and amide nitrogen resonances dictated by crystallographic symmetry (Figure 4), the markedly different chemical-shift dispersions within each nitrogen manifold reflect subtle yet systematic differences in local geometry and electronic structure. In particular, the broader ^15^N chemical-shift distribution observed for the Zn­(II) complex indicates a higher degree of coordination asymmetry, consistent with partial pendant arm coordination. By contrast, the more compact ^15^N chemical-shift pattern of the Cd­(II) complex reflects a more uniform coordination environment arising from full engagement of all four amide pendant arms. The ^15^N CP/MAS NMR spectrum of the free dotam ligand (Figure S10) provides an important reference for interpretation of the complex spectra. Two signals are observed in both amide and amine regions, consistent with the presence of two nonequivalent amine and amide groups in the crystallographic unit cell.^29,30^ Relative to this ligand reference, the spectra of the metal complexes show distinct metal-dependent changes in chemical shift and signal dispersion, demonstrating the sensitivity of ^15^N ss-NMR to coordination-induced changes in local geometry and electronic structure.

**Figure 4:**
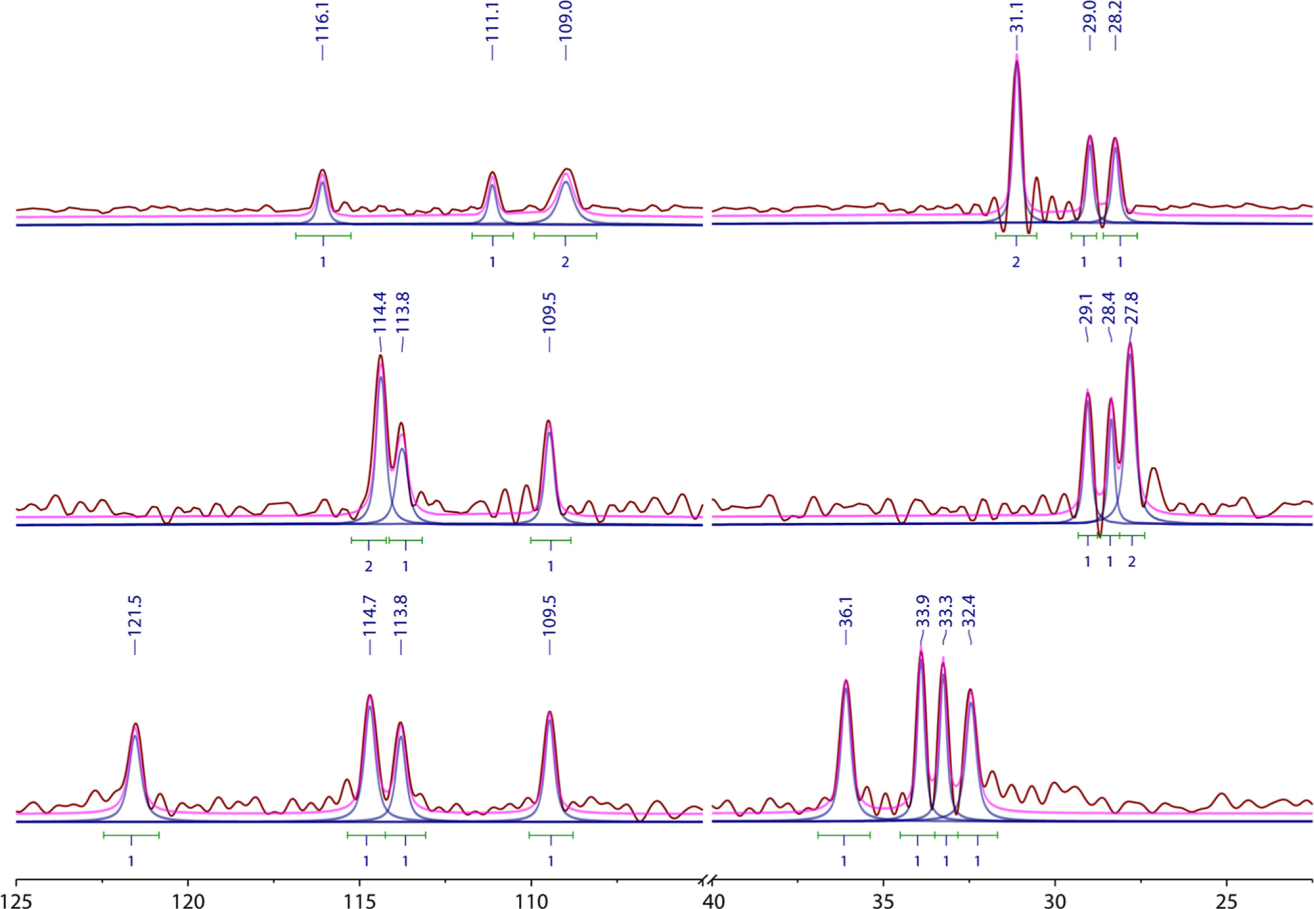
^15^N CP/MAS NMR spectra (51 MHz, 10 kHz) of [Zn­(dotam)]­[ZnCl_4_]·2.5H_2_O (top), [Cd­(dotam)]­[CdCl_4_]·0.5H_2_O (middle), and [Hg­(dotam)]­[Hg_3_Cl_8_]·3H_2_O (bottom). Color code: maroon: experimental spectra, blue: deconvoluted resonances of individual sites, magenta: sum of the deconvoluted resonances.

This spectroscopic sensitivity can be directly correlated with crystallographic metrics. In the Zn­(II) complex, the presence of both coordinated and noncoordinated pendant arms, combined with a pronounced displacement of the metal center from the N_4_ macrocyclic plane, leads to a wider distribution of M–N bond lengths and torsion angles, which is faithfully mirrored in the spread of both amine and amide ^15^N chemical shifts. In contrast, the Cd­(II) ion adopts a position closer to the centroidal axis between the N_4_ and O_4_ donor planes, resulting in a narrower range of M–N distances and a correspondingly reduced dispersion of ^15^N chemical shifts. Importantly, these distinctions are encoded in the local electronic environments of the nitrogen nuclei and are therefore directly observable by ss-NMR, even if the overall coordination polyhedra appear closely related in crystallographic terms. In addition, chemical shifts of the amine nitrogen nuclei are ∼5 ppm lower for both Zn­(II) and Cd­(II) complexes compared to that of the Hg­(II) complex. It can be attributed to a spin–orbit heavy atom-to-light atom effect of the Hg­(II) ion in the latter complex.^26,31^

The ability of ^15^N MAS NMR spectroscopy to resolve such differences highlights its role as a core component of an NMR crystallography approach, wherein experimental NMR observables and first-principles calculations provide complementary site-resolved information that extends beyond static atomic positions. Unlike solution NMR, where rapid molecular motion averages out inequivalent environments, ^15^N ss-NMR retains sensitivity to coordination asymmetry and metal-dependent bonding effects that are essential for understanding the origin of fluxional behavior observed in solution. The combined use of ^15^N ss-NMR, diffraction data, and GIPAW-DFT calculations thus enables a unified structural description linking solid-state geometry with solution dynamics, demonstrating the broader applicability of NMR crystallography to metal-ion complexes of macrocyclic ligands exhibiting subtle but functionally relevant coordination differences.

As a key step in the NMR crystallography analysis, the X-ray crystal structures were taken as starting models for GIPAW-DFT (CASTEP) calculations of ^13^C and ^15^N NMR shieldings. Linear regression of the calculated shieldings (Tables S10–S13) against experimental solid-state chemical shifts, using weighted averages for split carbonyl resonances, shows excellent correlations with slopes close to the theoretical value of −1.0 (Figure S11) and low root-mean-square deviations values. This confirms both the accuracy of the crystallographic models and the suitability of the computational approach. Importantly, this level of agreement is maintained despite partial accidental overlap of some experimental resonances, demonstrating that such an overlap does not compromise the robustness of the combined NMR–DFT analysis. The ss-NMR data remain sensitive to coordination number, pendant arm binding mode, and/or local coordination asymmetry, and it is reflected in site-specific chemical shifts and their dispersion. Consequently, NMR crystallography provides reliable, phase-specific local structural information that complements diffraction data and enables discrimination between closely related coordination environments that are not fully resolved by diffraction analysis alone.

### Variable-Temperature NMR Spectroscopy in Solution

To study the dynamics of the complexes in a water-containing solution, NMR spectra were measured in a temperature ranges of (−40)–60, (−40)–80 and (−10)–60 °C for the [Zn­(dotam)]^2+^, [Cd­(dotam)]^2+^ and [Hg­(dotam)]^2+^ complexes, respectively, with a 10 °C steps (the last complex has insufficient solubility below −10 °C). In this temperature range, a coalescence of two ^13^C NMR peaks assigned to the macrocycle carbon atoms was observed, analogously to the situation observed in other complexes of DOTA-like ligands.^24,32^ At ^13^C Larmor frequency of 101 MHz, the coalescence temperatures of approximately −20, 30, and 20 °C for the [Zn­(dotam)]^2+^, [Cd­(dotam)]^2+^ and [Hg­(dotam)]^2+^ complexes, respectively, were observed. To quantitatively understand dynamics of these systems, the interconversion rate constants, *k*_ex_, were calculated for temperatures below and above the coalescence by eqs 1 and 2, respectively, where *w* is a full width at half-maximum height of the exchanging signal, *w*_0_ is a full width of nonexchanging signal (in this case, the carbonyl ^13^C signal was used), and Δν is a difference in the chemical shifts of the exchanging signals in Hz.

1 $$k_{ex}=\pi \left(w-w_{0}\right)$$

2 $$k_{ex}=\frac{\pi \Delta \nu^{2}}{2\left(w-w_{0}\right)}$$

Afterward, an Eyring plot (plot of ln­(*k*_ex_/*T*) vs 1/*T*, Figure 5) was constructed and the experimental rate constants were fitted using a least-squares linear fitting, giving the activation enthalpy and activation entropy (Table 1) of the exchange process. From these parameters, it follows that the [Zn­(dotam)]^2+^ cation is more fluxional than the [Cd­(dotam)]^2+^ complex. For the estimation of the activation parameters only the exchange rates falling into a smooth temperature dependence were used (point measured at −40 °C was excluded for the Zn­(II) complex, and points measured below −10 °C were excluded for the Cd­(II) complex). Thus, Eyring plots remain linear, which makes the activation parameters sufficiently reliable despite the use of CD_3_OD as a cosolvent to prevent freezing of the solution.

**Figure 5:**
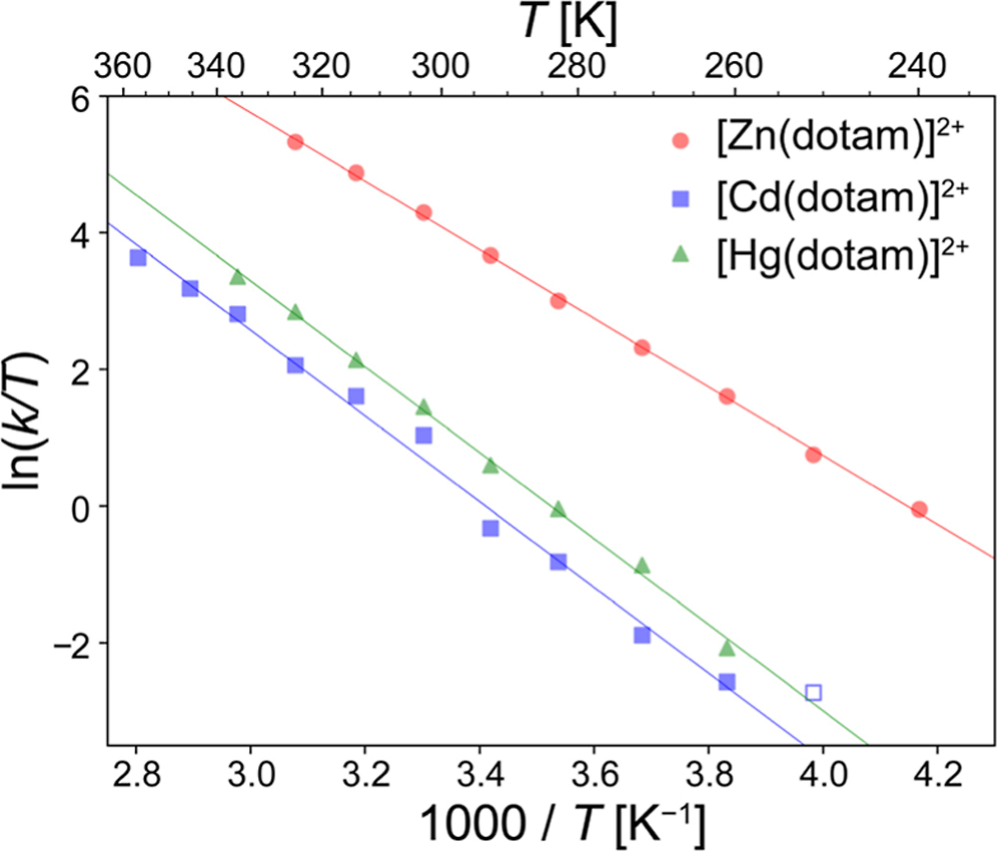
Eyring plot for isomerization rates of the title complexes in water or water-MeOH mixtures obtained by ^13^C­{^1^H} NMR spectra (at Larmor frequency 101 MHz). Points represent the experimental data, and the lines are the best least-squares fits. The hollow point was excluded from the fitting data set as an outlier.

**Table 1:** Activation Parameters of Isomer Interconversion for the [Zn­(dotam)]^2+^, [Cd­(dotam)]^2+^, and [Hg­(dotam)]^2+^ Complexes and Comparison with Those for the Corresponding DOTA Complexes (Italics, from ref 27)

compound	Δ*H* ^‡^ (kJ mol^–1^)	Δ*S* ^‡^ (J K^–1^ mol^–1^)	^298^Δ*G* ^‡^ (kJ mol^–1^)
[Zn(dotam)]^2+^	+41.7 ± 0.8	–24 ± 3	+49.0 ± 1.2
*[Zn(dota)]^2–^*	+37.0	–33	+47.0
[Cd(dotam)]^2+^	+52 ± 3	–20 ± 10	+58 ± 4
*[Cd(dota)]^2–^*	+60.6	+18	+55.1
[Hg(dotam)]^2+^	+52 ± 2	–13 ± 7	+56 ± 3
*[Hg(dota)]^2–^*	+53.6	–0.1	+54

## Discussion

### Solid-State Structures

During attempts to prepare [M­(dotam)]^2+^ (*M* = Zn, Cd) complexes as simple chloride salts, which were expected to be more suitable for ss-NMR measurements than the previously reported perchlorates,^24^ salts with tetrachlorometallate counter-anions were reproducibly formed instead. These salts crystallized preferentially, with optimal yields achieved at a MCl_2_/dotam molar ratio of 2:1. Their formation likely reflects a combination of favorable solid-state packing interactions between the large dicationic complexes and tetrahedral [MCl_4_]^2–^ anions and the relatively lower thermodynamic stability of the [M­(dotam)]^2+^ complexes compared to their DOTA analogues, leading to a competition between the macrocycle coordination and the chlorometallate formation in the solid state. Importantly, despite differences in counterions and crystal packing, the coordination polyhedra of the [M­(dotam)]^2+^ cations closely resemble those reported for complexes of the same metal ions with (dota)^4–^ as the ligand.^27^

The geometrical parameters of chlorometallate salts of the Zn­(II)– and Cd­(II)–dotam complexes are compared with those of the previously reported perchlorate analogues in Table 2. Overall, the coordination geometries are very similar: metal–nitrogen bond lengths, metal displacement from the N_4_ plane, and torsion angles along the pendant arms fall within a narrow range. The prepared chlorometallate salts exhibit slightly more regular M–O distances and pendant-arm torsion angles, most likely reflecting differences in crystal packing and lattice stabilization rather than intrinsic changes in the coordination mode of the [M­(dotam)]^2+^ cations.

**Table 2:** Comparison of selected bond lengths and angles of [M­(dotam)]^2+^ complexes with previously published structures

parameter	[Zn(dotam)][ZnCl_4_]·2.5H_2_O	[Zn(dotam)](ClO_4_)_2_·H_2_O^24^	[Cd(dotam)][CdCl_4_]·0.5H_2_O	[Cd(dotam)](ClO_4_)_2_·1.5H_2_O^24^
distances, Å				
M-O1	3.283(1)	3.304	2.500(1)	2.633
M-O2	2.080(1)	2.162	2.371(1)	2.322
M-O3	2.995(1)	3.062	2.584(1)	2.648
M-O4	2.085(1)	2.036	2.322(1)	2.361
M-N1	2.242(1)	2.220	2.435(2)	2.468
M-N2	2.226(1)	2.204	2.411(2)	2.443
M-N3	2.250(1)	2.253	2.446(2)	2.431
M-N4	2.266(1)	2.311	2.457(2)	2.416
M-Q_N_ ^a^	0.939	0.946	1.246	1.263
M-Q_O_ ^a^	1.534	1.558	1.322	1.319
angles, °				
N1-Q_N_-Q_O_-O1^a^	28.31	32.67	25.45	25.64
N2-Q_N_-Q_O_-O2^a^	25.91	22.92	26.81	24.33
N3-Q_N_-Q_O_-O3^a^	25.46	24.42	24.49	23.82
N4-Q_N_-Q_O_-O4^a^	25.81	22.98	26.19	23.85

a Q_N_ is the centroid of the N_4_ plane; Q_O_ is a centroid of two coordinated O atoms for Zn­(II) complexes and four coordinated O atoms for Cd­(II) complexes.

### Dynamics in Solution

Values of the activation parameters (Table 1) show that the [Zn­(dotam)]^2+^ cation is more fluxional than the [Cd­(dotam)]^2+^ and [Hg­(dotam)]^2+^ complexes. This finding is consistent with the solid-state structures where the Zn­(II) ion is coordinated only by two pendant arms, with the other two pendant arms being nonbound. These two pairs of pendant arms can quickly mutually interchange in solution, resulting in the averaging of pendant methylene signals into a single peak. In the Cd­(II)/Hg­(II) complexes, all four pendant arms are coordinated, resulting in a more rigid coordination environment and a correspondingly higher activation barrier for the isomer interconversion. In the isomerization mechanism consistent with the data, the progressive weakening and reorganization of metal ion–oxygen interactions leads to all pendant arms participating simultaneously in a symmetric, high-coordination-number transient state. It is supported by uniformly negative activation entropy observed for all [M­(dotam)]^2+^ complexes, suggesting the involvement of a more ordered high-symmetry transition state. Such a transition state can be rationalized by transient engagement of all four amide pendant arms in approximately *C*_4_-symmetric square-antiprismatic geometry. Access to this configuration is facilitated by the weaker and more labile metal ion–amide oxygen interactions compared to the stronger anionic metal ion–carboxylate bonds in the DOTA complexes.^27^ The neutral character of the amide group further reduces electrostatic penalties associated with the simultaneous pendant arm coordination, promoting cooperative rather than stepwise rearrangement of the pendant arms. This effect is expected to be particularly pronounced for the heavier and softer metal ions Cd­(II) and Hg­(II), for which softer donor atoms and longer metal ion–oxygen bonds lower the energetic cost of reorganizing the coordination sphere. Consistent with this interpretation, the [Cd­(dotam)]^2+^ complex exhibits a lower activation enthalpy than its DOTA analogue, supporting a mechanism that proceeds without complete bond dissociation. This observation is consistent with solution-state dynamics observed for another soft metal ion complex, [Pb­(dotam)]^2+^ cation, in which a symmetrical square-prismatic intermediate was suggested.^29^

As demonstrated above, the solution dynamics of the dotam and DOTA complexes differ markedly despite close similarities in solid-state geometry. For the [M­(dotam)]^2+^ series, there is a common underlying mechanism of isomer interconversion across the entire metal series. In contrast, the corresponding [M­(dota)]^2–^ complexes display a metal ion-dependent behavior.^27^ Taken together, these observations demonstrate that even subtle changes in pendant-arm donor type and charge profoundly affect solution dynamics, despite only minor differences in static solid-state structures. The isomer interconversion of the dotam complexes is entropy-controlled via a symmetric transition state enabled by the weak and flexible metal ion–amide group interactions. The dotam complexes thus provide a clear example of how weak and flexible metal ion–ligand interactions can enforce this isomerization pathway. Isomerization of the DOTA complexes exhibits metal ion-specific associative or dissociative pathways, dictated by the stronger metal ion–carboxylate anion interaction. The replacement of the hard acetate pendant arms by the softer *O*-coordinated acetamide pendant arms leads to a change of the mechanism of the isomer interconversion for the particular metal ion.

### ^13^C–^14^N Coupling as a Site-Specific Probe of Amide Coordination

The ss-NMR spectra are fully consistent with the crystallographic models, as evidenced by the number of resolved resonances, the low root-mean-square deviations between experimental chemical shifts and GIPAW-DFT-calculated shielding, and the near-ideal linear correlations between the calculated shielding and the experimental shifts (slope approximately −1.0; Table 3). Together, these metrics confirm both the phase purity of the samples and the reliability of the combined XRD–NMR–DFT approach. Despite this overall agreement, an unexpected feature emerges in the carbonyl region of the ^13^C CP/MAS spectra where several resonances appear as asymmetric doublets. For the Zn­(II) complex, such splitting could, in principle, be attributed to crystallographic disorder affecting two of the pendant arms. However, this explanation cannot account for this behavior observed for the Cd­(II) complex, which exhibits no crystallographic disorder yet displays splitting for all four carbonyl carbon signals. This unequivocally rules out static structural disorder as the origin of the observed splitting. Therefore, we attribute the carbonyl peak splitting to residual heteronuclear ^13^C–^14^N scalar and dipolar interactions between the carbonyl carbon nuclei (spin 1/2) and the directly bonded amide ^14^N nuclei (spin 1).^28^ Such couplings are known to become observable when quadrupolar relaxation of the ^14^N nucleus is sufficiently reduced. Importantly, the occurrence of the resolved doublets directly correlates with the metal ion coordination by the amide groups. In the Zn­(II) complex, only two carbonyl resonances are split, thus identifying these sites as metal ion-coordinated amide groups. This assignment is further supported by the GIPAW-DFT.

**Table 3:** Root-Mean-Squared Deviations (in ppm) of the Linear Regressions of the Predicted Against Experimental NMR Parameters of the Investigated Compounds and Nuclei

compound	^13^C	^15^N
[Zn(dotam)][ZnCl_4_]·2.5H_2_O	0.38	1.81
[Cd(dotam)][CdCl_4_]·0.5H_2_O	0.55	0.56
[Hg(dotam)][Hg_3_Cl_8_]·3H_2_O^26^	0.57	8.58

In the Cd­(II) complex, all four amide carbonyl groups participate in metal ion coordination and, consequently, all four ^13^C carbonyl resonances exhibit resolved splitting. Moreover, the magnitude of the residual coupling is sensitive to subtle variations in coordination strength: carbonyl groups associated with the longer Cd–O bond distances (Table S4) display smaller apparent coupling constants (∼26 Hz) compared to those with shorter Cd–O contacts (∼32–35 Hz). This trend indicates that weaker metal ion–oxygen interactions reduce the perturbation of the amide nitrogen electric field gradient, thereby diminishing the extent to which the ^13^C–^14^N coupling is retained under MAS conditions.

This effect can also be observed in the ^13^C ss-NMR spectrum of the Hg­(II) complex, where a more significant difference between the metal-to-carbonyl oxygen atom distances is observed compared to that present in the Cd­(II) complex. Consequently, only the carbonyl peaks of two more strongly bound carbonyl groups are observed as doublets.^26^

The sensitivity of ss-NMR spectra to subtle changes in the amide group coordination can be illustrated by the dependence of value of the residual dipolar coupling on M–O bond valence calculated from X-ray diffraction M–O distances in all three metal complexes according to the model suggested by Brown & Altermatt (Figure 6).^33–35^ The splitting is clearly increasing with the stronger oxygen donor-metal ion interaction and, thus, its value can be used to evaluate the relative strength of oxygen atom-metal ion interaction in coordinated amide groups in the solid state. This ss-NMR information is complementary to data from diffraction methods.

**Figure 6:**
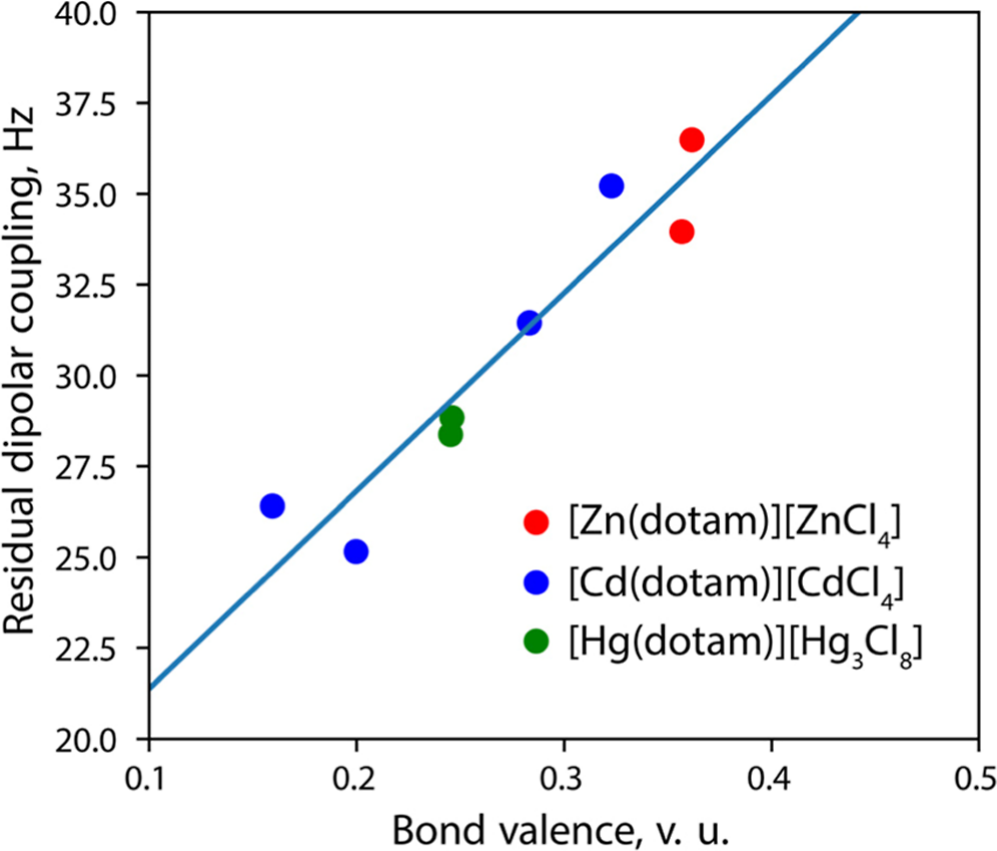
Dependence (R.D.C = 16 + 54·B.V.; *R* ^2^ = 0.9) of the residual ^13^C–^14^N dipolar coupling of carbonyl resonance on CO···M coordination bond valence (in v. *u*. = valence units). The points represent the experimental residual couplings, and the line shows the best linear regression fit.

In contrast, no such splitting is observed in the carbonyl regions of the corresponding DOTA complexes,^27^ which adopt comparable solid-state geometries but lack directly bonded amide nitrogen atoms. This comparison conclusively demonstrates that the observed peak doubling originates from interactions with the amidic ^14^N nuclei rather than from more distant macrocyclic nitrogen atoms or purely carbonyl-centered effects.

Taken together, these findings establish the resolved ^13^C–^14^N splitting as a sensitive, site-specific spectroscopic marker of the amide coordination and metal ion–ligand bonding strength in dotam complexes. More broadly, the data highlight the ability of ss-NMR to reveal local electronic and quadrupolar effects that are invisible to diffraction methods and averaged out in solution, reinforcing the central role of NMR crystallography in elucidating subtle coordination phenomena in metal ion complexes of macrocyclic ligands.

Importantly, the residual ^13^C–^14^N splitting observed here goes beyond a system-specific spectroscopic peculiarity and represents a structurally meaningful diagnostic parameter. Unlike conventional chemical shifts, which reflect averaged electronic environments, the partially retained ^13^C–^14^N dipolar interaction directly reports on the local electric field gradient at the amide nitrogen nucleus and, therefore, on the strength and asymmetry of metal–amide coordination. The magnitude and presence of the splitting unambiguously differentiate coordinated and noncoordinated amide groups and quantitatively track variations in metal ion–oxygen bond strength within a single complex. This establishes residual ^13^C–^14^N coupling as a site-resolved probe of amide coordination that is sensitive to subtle bonding differences not reliably distinguishable by diffraction metrics alone. Given the widespread use of amide-functionalized macrocycles and chelators in coordination chemistry and bioinorganic applications, this effect may serve as a generally applicable solid-state NMR marker to assign amide donor engagement and relative coordination strength in metal complexes. In this sense, the present findings extend the scope of NMR crystallography by demonstrating that quadrupolar-mediated residual heteronuclear couplings can provide direct structural information on metal ion–ligand bonding interactions in molecular solids.

## Conclusion

The present study provides a comprehensive view of dotam complexes with d^10^ metal ions by linking the solid-state structure, local electronic environments, and solution dynamics within a unified experimental and computational framework. Although coordination polyhedra of the Zn­(II), Cd­(II), and Hg­(II) complexes appear closely related by X-ray diffraction, the multinuclear ss-NMR spectroscopy reveals pronounced metal-dependent differences in coordination asymmetry and pendant arm binding. These differences persist in the solid state but are averaged out in solution. These static features rationalize the distinct dynamic behavior observed by variable-temperature solution-state NMR spectroscopy and underline the importance of weak metal ion–amide group interactions in governing isomer interconversion mechanisms. The most interesting observation of the study, the dependence of the residual ^13^C–^14^N dipolar coupling on subtle changes of the amide coordination to metal ions, could possibly be used for a general evaluation of amide coordination in the solid complexes by ss-NMR. Overall, these results highlight NMR crystallography as a uniquely powerful approach that combines ss-NMR spectroscopy and first-principles calculations to access site-specific coordination asymmetry and metal ion–ligand bonding effects that remain hidden to diffraction techniques and are averaged out in solution.

## Experimental Section

### Safety Considerations

Compounds containing Cd­(II) and Hg­(II) were handled as toxic heavy metal materials using standard precautions for hazardous chemicals, including appropriate personal protective equipment and manipulation in a fume hood. Thermogravimetric analysis showed that the investigated complexes are thermally stable and do not exhibit an unexpected release of heavy metals under the applied conditions. Waste containing Cd or Hg was collected separately and disposed of according to institutional and statutory regulations for hazardous heavy metal waste.

### General

Chemicals and solvents were purchased from common vendors with an analytical purity. Ligand dotam was obtained from Chematech (France). The Hg­(II) complex of dotam was prepared as previously described.^26^ Mass spectra were recorded using a Waters ACQUITY QDa MS detector (part of the Waters Arc HPLC system) through a direct inlet. Samples were dissolved in 0.1% TFA/water mixture, and the solution was inserted into a mass spectrometer. The MS data were processed by using Empower 3 software. Elemental analyses were carried out at the IOCB (Prague) by combustion analysis.

Diffraction data were collected at 120 K (Cryostream Cooler, Oxford Cryosystem) on a Bruker D8 VENTURE Kappa Duo PHOTON100 diffractometer with an IμS microfocus-sealed tube using Mo-*K*α (λ = 0.71073 Å) radiation. Data were analyzed using the SAINT (Bruker AXS Inc.) software package and subsequently corrected for absorption effects using numerical method (SADABS). The structures were solved using direct methods (SHELXT2018/2)^36^ and refined with full-matrix least-squares techniques (SHELXL2019/2).^37^ All non-hydrogen atoms were refined anisotropically. All hydrogen atoms were found in the difference electron density map. However, hydrogen atoms bound to carbon atoms were fixed in theoretical positions using *U*_eq_(H) = 1.2 *U*_eq_(C) to keep the number of fitted parameters low, and only hydrogen atoms bound to oxygen atoms were tried to fully refine. However, some heteroatom-bound hydrogen atoms were fixed in the original positions, as the geometry during the refinement was unstable and heteroatom–hydrogen bond distances became unrealistically long or short. In the [Zn­(dotam)]­[ZnCl_4_]·2.5H_2_O, the disordered amidic nitrogen atoms were refined over two sites with approximately 0.6:0.4 occupancy parameters, their ADPs were made equal using the EADP command, and the amidic hydrogen atoms were refined as bound to both disordered nitrogen atoms.

Solution ^1^H and ^13^C­{^1^H} NMR spectra were measured on Bruker Avance III Neo 400 (resonance frequencies 400/101 MHz for ^1^H/^13^C, respectively) or Varian Unity Inova 400 (VT experiments). All NMR spectra were acquired at 25 °C unless stated otherwise. Spectra were referenced on ^1^H and ^13^C NMR signals of the *t*BuOH methyl group (1.24/30.29 ppm, respectively). For temperature calibration, a standalone sealed NMR tube containing 80% ethylene glycol in dmso-*d*_6_ (for temperatures higher than 20 °C) or 99.8% MeOH-*d*_4_ (for temperatures below 20 °C) was used. For the VT experiments, the metal complexes (ca. 20 mg) were dissolved/suspended in D_2_O (0.5 mL, containing 0.1% v/v *t*BuOH). For the low temperature experiments, up to 40% v/v MeOH-*d*_4_ was added to prevent freezing of the solution. Prior to each acquisition, the sample was left to equilibrate at the chosen temperature for at least 15 min. Then, the D_2_O deuterium signal was locked, the spectrometer was shimmed, and ^13^C­{^1^H} NMR spectra were acquired. The ^13^C­{^1^H} spectra were referenced so that the pendant CH_2_ resonance stayed at a constant chemical shift throughout the temperature series. This ensured that the high-temperature shift of the macrocycle CH_2_ groups fell halfway between the low-temperature peaks. The spectra were then phase- and baseline-corrected. For each peak, its position and width at half-maximum height were obtained by line-shape analysis in MestReNova. The ^113^Cd NMR spectrum was measured on Bruker Avance III 600 (resonance frequency 133.1 MHz). The ^199^Hg NMR spectrum of the [Hg­(dotam)]^2+^ cation was measured with a perchlorate (prepared according to the literature^25^) as the counteranion (due to too low solubility of salts with the metalate anions) on a Bruker Avance III Neo 400 (resonance frequency 71.5 MHz). The ^113^Cd and ^199^Hg NMR spectra were referenced using a unified Ξ_*i*_ scale to avoid use of toxic primary references.

The solid-state ^13^C­{^1^H} and ^15^N­{^1^H} CP-ss-NMR spectra were obtained using a Bruker Avance III HD 500 NMR spectrometer (^13^C resonance frequency 126 MHz, ^15^N resonance frequency 51 MHz) equipped with a 4 mm broad-band probe under MAS frequency 10 kHz. The ^13^C and ^15^N NMR scale was calibrated with glycine as an external standard (δ_C_ = 176.03 ppm for the low-field carbonyl signal, δ_N_ = 34.35 ppm). The ^1^H DUMBO ss-NMR spectra were measured on a Bruker Avance NEO 700 MHz spectrometer equipped with a 3.2 mm broad-band probe under MAS frequency 10 kHz. All spectra were Fourier transformed, phase- and baseline-corrected using MestReNova software. To improve resolution of the ^13^C and ^15^N ss- CP-NMR spectra, the FIDs were zero-filled to 16k points. Spectra were deconvoluted in MestReNova to obtain chemical shifts, line widths, and areas under the peaks using *L*/*G* = 0.8 as a default parameter for all peaks.

The unit cell parameters were kept fixed, and all internal coordinates were subject to optimization with respect to the crystal-lattice energy by the PW DFT (plane-waves density-functional theory) implementation in the CASTEP code.^38–40^ For the disordered atoms, only the major part of the disorder was kept. The PBE^41^ functional was applied together with the ZORA (scalar-relativistic zeroth-order regular approximation) scheme^42^ and with the “Fine” level of settings of the CASTEP version 16.1. In particular, the PW cutoff value was 571 eV and the Monkhorst–Pack grids^43^ to sample the Brillouin zone were 2 × 1 × 2 (no offset; 2 k-points) in the case of [Cd­(dotam)]­[CdCl_4_]·0.5H_2_O, and 3 × 2 × 1 (no offset; 2 *k*-points) in the case of [Zn­(dotam)]­[ZnCl_4_]·2.5H_2_O. The optimized structures were then used to predict the NMR shielding of the ^13^C and ^15^N nuclei. The same PBE-ZORA approach as employed in geometry optimizations was combined with the gauge-including projector augmented wave (GIPAW) method in order to predict the NMR chemical shielding tensors of all nuclei.^44,45^ The CASTEP-NMR module^40^ was used.

### Synthesis of [M­(dotam)]­[MCl_4_]·*x*H_2_O (*M* = Zn, *x* = 2.5 and *M* = Cd, *x* = 0.5)

The ligand dotam (0.50 g, 1.3 mmol) was weighted into a 100 ml round-bottom flask and dissolved in H_2_O (15 mL) and MeOH (45 mL). Then, MCl_2_ (2.5 equiv.; 0.43 g of ZnCl_2_ or 0.71 g of CdCl_2_·2.5H_2_O) was added and the solution was stirred at 75 °C overnight. Afterward, the solvent was removed in vacuo. The residue was dissolved in water, and the solution was heated by a heat gun until the solid dissolved. The hot solution was left to freely cool down to room temperature and then it was kept in a refrigerator. After several days, a clear crystalline product was collected by filtration, washed with acetone, Et_2_O, and dried in air, yielding [Zn­(dotam)]­[ZnCl_4_]·2.5H_2_O (0.79 g, 87%) or [Cd­(dotam)]­[CdCl_4_]·0.5H_2_O (0.55 g, 58%) as clear single-phase crystalline solids.

[Zn­(dotam)]­[ZnCl_4_]·2.5H_2_O. ^1^H NMR (D_2_O, 400 MHz): 3.54 (8H, s, NCH_2_CONH_2_), 3.10–3.00 (8H, m, ring CH_2_), 2.87–2.77 (8H, m, ring CH_2_); ^13^C­{^1^H} (D_2_O, 101 MHz): 175.3 (*C*O), 55.6 (*C*H_2_CONH_2_), 51.1 (ring CH_2_). ESI MS: (+) 463.22 (463.18, {[Zn­(dotam)]^2+^–H^+^}^+^). Elemental analysis: anal. (calcd. for [Zn­(dotam)]­[ZnCl_4_]·2.5H_2_O): C: 26.84 (26.76) 4.74 (5.19) 15.29 (15.60).

[Cd­(dotam)]­[CdCl_4_]·0.5H_2_O. ^1^H NMR (D_2_O, 400 MHz): 3.38 (8H, s, NCH_2_CONH_2_), 2.94 (8H, s, ring CH_2_), 2.64 (8H, s, ring CH_2_); ^13^C­{^1^H} (D_2_O, 101 MHz): 176.2 (CO), 55.0 (CH_2_CONH_2_), 51.5 (ring CH_2_), 48.2 (ring CH_2_); ^113^Cd (D_2_O, 133 MHz): −553 (s, [CdCl_4_]^2–^), −565 ([Cd­(dotam)]^2+^). ESI MS: (+) 513.18 (513.15, {[Cd­(dotam)]^2+^–H^+^}^+^). Elemental analysis: anal. (calcd. for [Cd­(dotam)]­[CdCl_4_]·0.5H_2_O): C: 24.99 (25.02) H: 3.92 (3.28) N: 14.41 (14.59).

### NMR Characterization Data of [Hg­(dotam)]­(ClO_4_)_2_

^1^H NMR (D_2_O, 400 MHz): 3.45 (8H, s, NCH_2_CONH_2_), 2.99 (8H, s, ring CH_2_), 2.80–2.63 (8H, m, ring CH_2_); ^13^C­{^1^H} (D_2_O, 101 MHz): 175.1 (*C*O), 54.2 (*C*H_2_CONH_2_), 51.5–45.6 (ring CH_2_, broad). ^199^Hg (D_2_O, 72 MHz): −1816 (s).

## Supplementary Material



## Notes

Experimental X-ray diffraction data, crystal packing information, additional bond distances and angles, solution NMR spectra, ^1^H ss-NMR spectra, additional information on ^13^C and ^15^N ss-NMR spectra, tables characterizing fitting of ^13^C and ^15^N ss-NMR spectra, DFT calculations of ^13^C and ^15^N ss-NMR shifts and shieldings, and plots of ^13^C and ^15^N experimental shift vs calculated shielding (PDF)
