# Supplementary material for: Complexes of DOTAM with d10 Divalent Metal Ions: X‑ray Diffraction and NMR Studies in Solution and the Solid State
Source: Inorg Chem. 2026 May 28;65(22):12398–408. doi: 10.1021/acs.inorgchem.6c01226 (PMC13250988; doi:10.1021/acs.inorgchem.6c01226)
Supplement: Supplementary file 1 [file ic6c01226_si_001.pdf]

SUPPORTING INFORMATION

for

Complexes of DOTAM with d^{10} Divalent Metal Ions: X-ray Diffraction, and NMR Studies in Solution and the Solid State

Jakub Obuch[a,b], Ivana Císařová[a], Jiří Czernek[b], Jiří Brus[b]* and Petr Hermann[a]*

[a]Department of Inorganic Chemistry, Faculty of Science, Charles University, Hlavova 2030/8, 128 40 Prague 2, Czech Republic. E-mail: petrh@natur.cuni.cz

[b]Institute of Macromolecular Chemistry, Czech Academy of Science, Heyrovského náměstí 2, 162 00 Prague 6, Czech Republic. E-mail: brus@imc.cas.cz

Content:

X-ray diffraction studies S2

Solution NMR spectroscopy S9

Solid-state NMR spectroscopy S11

X-ray diffraction studies

Table S1. Experimental X-ray diffraction data and details of the structure determination.

Parameter	$[C_{16}H_{32}N_8O_4Zn][ZnCl_4]\cdot 2.5H_2O$	$[C_{16}H_{32}CdN_8O_4][CdCl_4]\cdot 0.5H_2O$
Formula weight	718.07	776.10
Crystal system	monoclinic	monoclinic
Space group	$P2_1/c$ (No. 14)	$P2_1/n$ (No. 14)
a, *b*, *c* [Å]	8.6953(3), 14.0881(4), 23.2148(8)	10.2320(6), 19.0506(13), 14.1459(9)
α, β, γ [°]	90, 95.140(1), 90	90, 90.457(2), 90
V [Å^3]	2832.38(16)	2757.3(3)
Z	4	4
D(calc) [g cm^{-3}]	1.684	1.870
μ [mm^{-1}]	2.119	1.970
F(000)	1476	1540
Crystal size [mm]	0.18 × 0.22 × 0.26	0.13 × 0.17 × 0.19
Temperature [K]	120	120
Radiation [Å]	Mo-Kα, 0.71073	Mo-Kα, 0.71073
θ Min, Max [°]	2.3, 27.5	2.1, 27.5
h; *k*; *l* indices	−11:10; −17:18; −30:30	−13:13; −22:24; −17:18
Total / Unique data, R(int)	46792 / 6508, 0.023	34509 / 6326, 0.020
Observed data	6234	6178
N_{ref}, N_{par}	6508, 387	6326, 351
R, wR_2, S	0.0191, 0.0468, 1.07	0.0170, 0.0392, 1.19
Min./Max. resid. elect. density [e Å^{-3}]	−0.34 / 0.37	−0.33 / 0.45
CCDC number	2532330	2532331

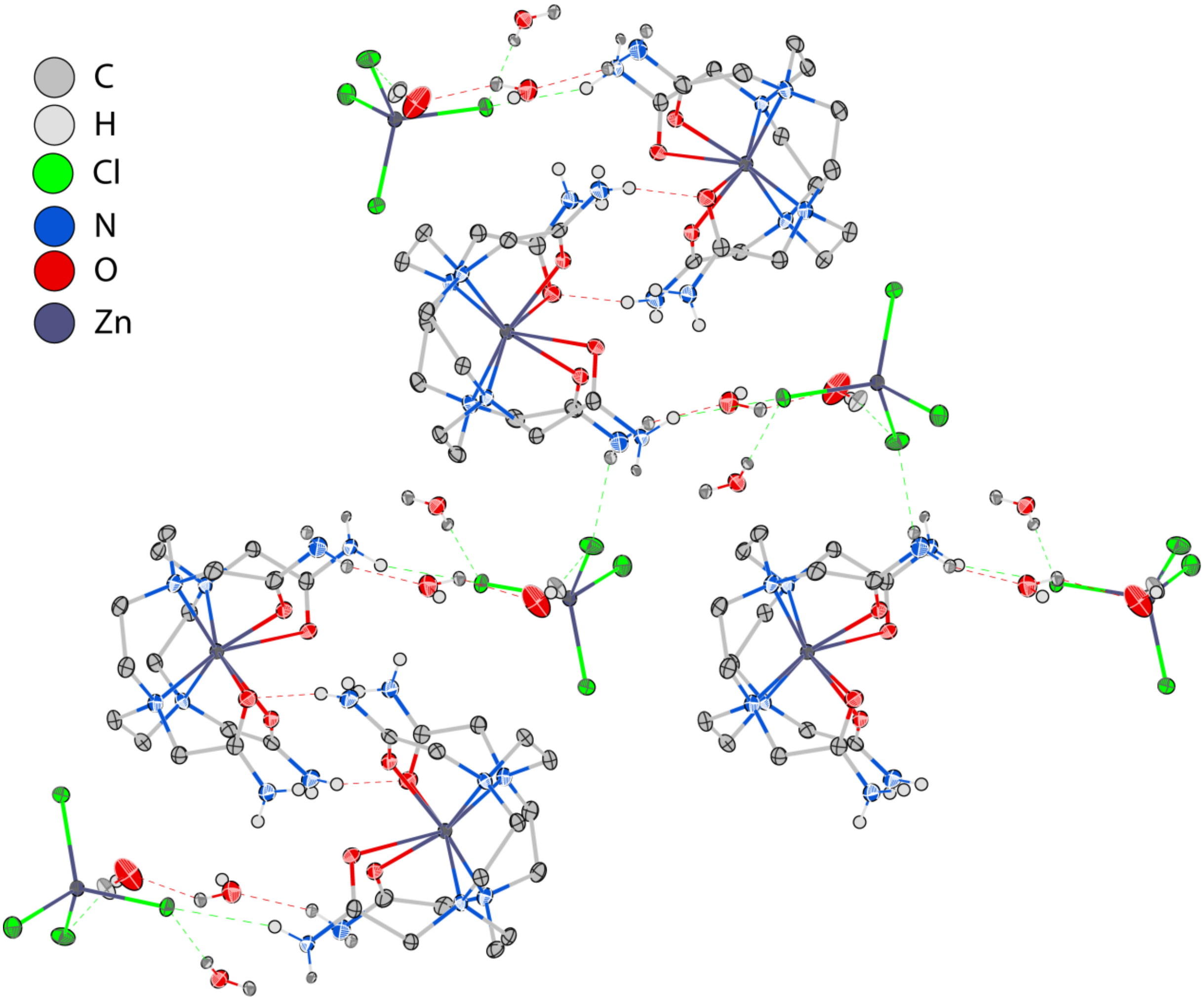

Figure S1. View along the axis *a* of the crystal packing in the structure of [Zn(**dotam**)][$ZnCl_4$]·2.5H_2O. Carbon-bound hydrogen atoms and the minor parts of the disorders are omitted for clarity. Dashed bonds represent hydrogen bonds. Thermal ellipsoids are drawn at 50 % probability level.

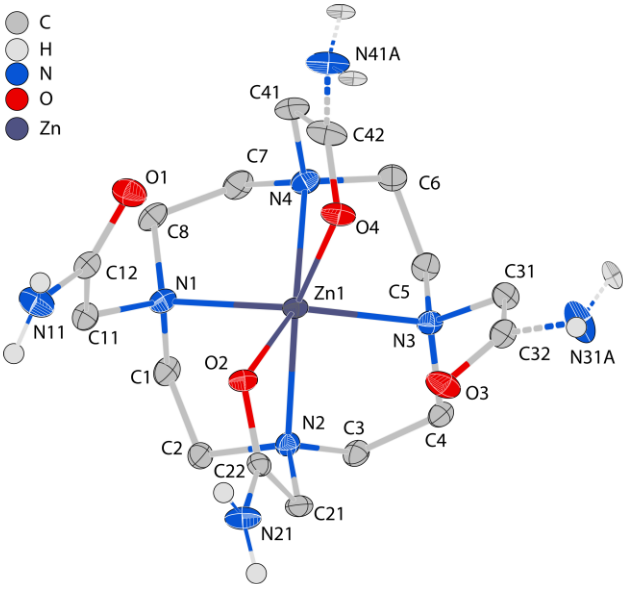

Figure S2. View of the complex [Zn(**dotam**)]$^{2+}$ cation in the structure of [Zn(**dotam**)][$ZnCl_4$]·2.5H_2O with atom labelling necessary for the following discussion.

Table S2. Selected geometrical parameters in the structure of [Zn(**dotam**)][$ZnCl_4$]·2.5H_2O.

Distances, Å			
Zn1–O1	3.2834(10)	Zn1–N1	2.2422(12)
Zn1–O2	2.0804(10)	Zn1–N2	2.2260(11)
Zn1–O3	2.9954(10)	Zn1–N3	2.2497(12)
Zn1–O4	2.0854(10)	Zn1–N4	2.2664(12)
Zn1–Q_{O2}	1.534	Zn1–Q_N	0.939
Zn2–Cl1	2.2248(6)	Zn2–Cl3	2.3012(5)
Zn2–Cl2	2.2863(4)	Zn2–Cl4A	2.316(3)
Zn2–Cl4B	2.227(2)		
Angles, °			
N1–Zn1–N2	81.47(4)	N1–Zn1–O4	122.00(4)
N1–Zn1–N3	132.11(4)	N2–Zn1–O2	76.51(4)
N1–Zn1–N4	78.84(4)	N2–Zn1–O4	150.64(4)
N2–Zn1–N3	79.57(4)	N3–Zn1–O2	126.07(4)
N2–Zn1–N4	129.07(4)	N3–Zn1–O4	93.53(4)
N3–Zn1–N4	79.93(4)	N4–Zn1–O2	149.38(4)
O2–Zn1–O4	85.12(4)	N4–Zn1–O4	76.54(4)
N1–Zn1–O2	90.81(4)		
Cl1–Zn2–Cl2	113.17(2)	Cl2–Zn2–Cl4A	102.82(15)
Cl1–Zn2–Cl3	108.02(2)	Cl2–Zn2–Cl4B	115.2(3)
Cl1–Zn2–Cl4A	113.30(5)	Cl3–Zn2–Cl4A	113.32(13)
Cl1–Zn2–Cl4B	110.27(9)	Cl3–Zn2–Cl4B	103.4(2)
Cl2–Zn2–Cl3	106.01(2)		
Torsion angles, °			
N1–Q_N–Q_{O2}–O1 [a]	−28.31	N3–Q_N–Q_{O2}–O3 [a]	−25.46
N2–Q_N–Q_{O2}–O2 [a]	−25.91	N4–Q_N–Q_{O2}–O4 [a]	−25.81

[a] Q_N is the centroid of the N_4 plane; Q_{2O} is a centroid of two coordinated O atoms.

Table S3. Hydrogen bonds in the structure of [Zn(**dotam**)][$ZnCl_4$]·2.5H_2O.

D—H···A	D—H	H···A	D···A	∠D-H-A
O1W—H1X···$Cl2^{i}$	0.76(3)	2.52(3)	3.2705(15)	167(3)
O1W—H1Y···Cl3	0.83(3)	2.41(3)	3.2144(15)	165(3)
O2W—H2E···O1W	0.82	2.26	2.682(3)	112
O2W—H2F···O1W	0.78	2.12	2.682(3)	129
O3W—H3X···N31A	0.77	2.47	2.981(7)	126
O3W—H3X···$Cl2^{ii}$	0.77	2.67	3.370(2)	152
O3W—H3Y···Cl4A	0.76	2.5	3.262(6)	179
O3W—H3Y···Cl4B	0.76	2.13	2.885(8)	170
N11—H11C···$O2W^{iii}$	0.83(2)	2.19(2)	3.016(3)	174.5(18)
N11—H11C···$O2V^{iii}$	0.83(2)	2.29(2)	3.093(3)	165.3(17)
N11—H11D···$O2^{iii}$	0.78(2)	2.24(2)	3.0040(15)	166(2)
N21—H21C···$O1^{iii}$	0.858(19)	2.048(19)	2.8888(16)	166.2(18)
N21—H21D···$O3^{iv}$	0.822(18)	2.162(18)	2.8210(16)	137.3(17)
N31B—H31C···$Cl2^{ii}$	0.87(2)	2.80(2)	3.616(4)	156.4(16)
N31B—H31D···Cl4A	0.86(2)	2.50(2)	3.339(3)	164.6(18)
N31B—H31D···Cl4B	0.86(2)	2.49(2)	3.335(4)	168.3(19)
N41B—H41C···$Cl3^{v}$	0.84(2)	2.48(2)	3.315(4)	177.3(17)
N41B—H41D···O2W	0.86(2)	1.99(2)	2.831(5)	165.9(17)
N41B—H41D···O2V	0.86(2)	2.30(2)	3.162(5)	175.1(19)

Symmetry codes: i: $1 + x, y, z$, ii: $-x, 1/2 + y, 1/2 - z$, iii: $1 - x, 1 - y, 1 - z$, iv: $-x, 1 - y, 1 - z$, v: $1 - x, 1/2 + y, 1/2 - z$.

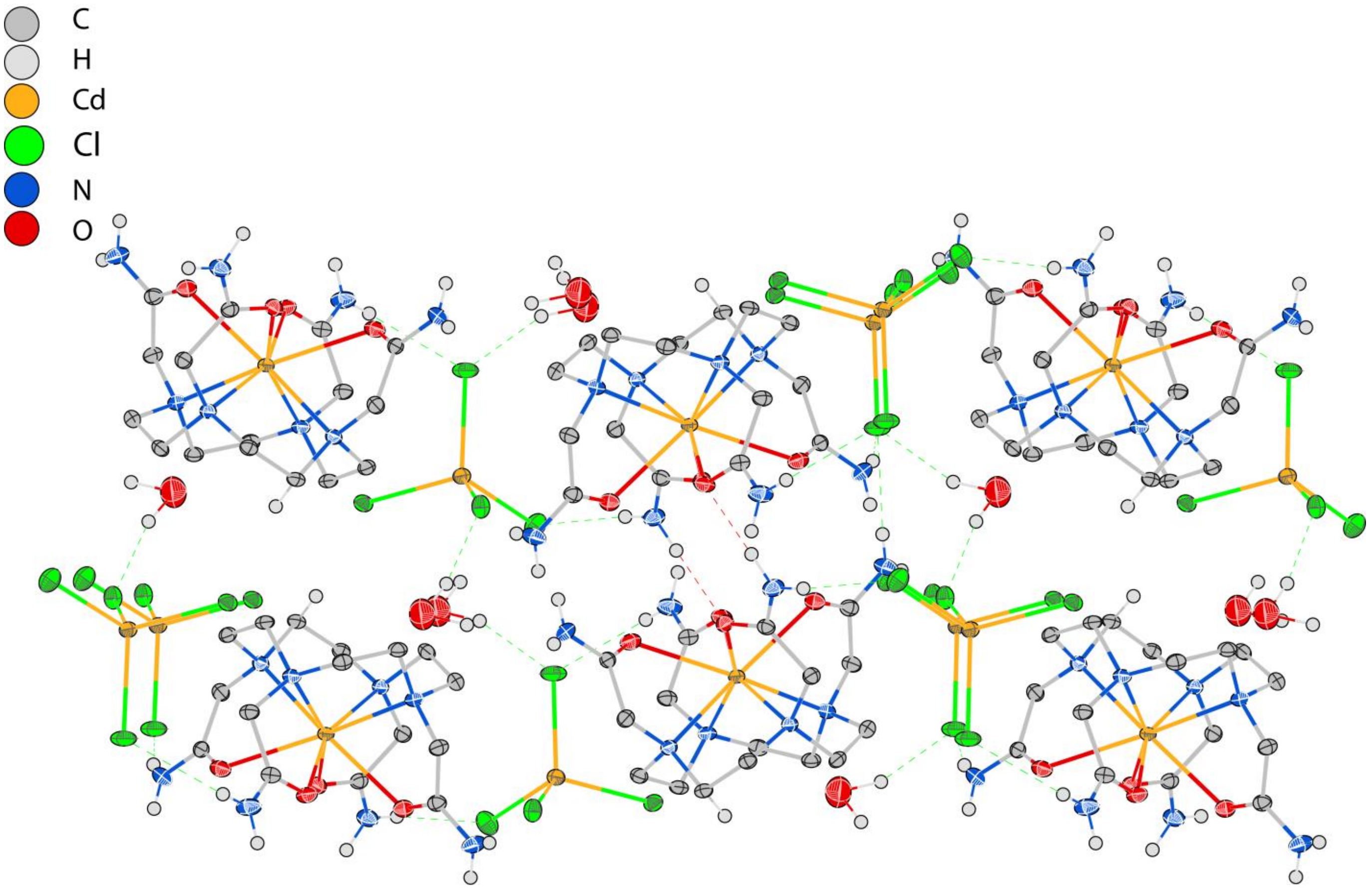

Figure S3. View along the axis *a* of the crystal packing in the structure of [Cd(**dotam**)][$CdCl_4$]·0.5H_2O. Carbon-bound hydrogen atoms and minor parts of the disorders are omitted for clarity. Dashed bonds represent hydrogen bonds. Thermal ellipsoids are drawn at 50 % probability level.

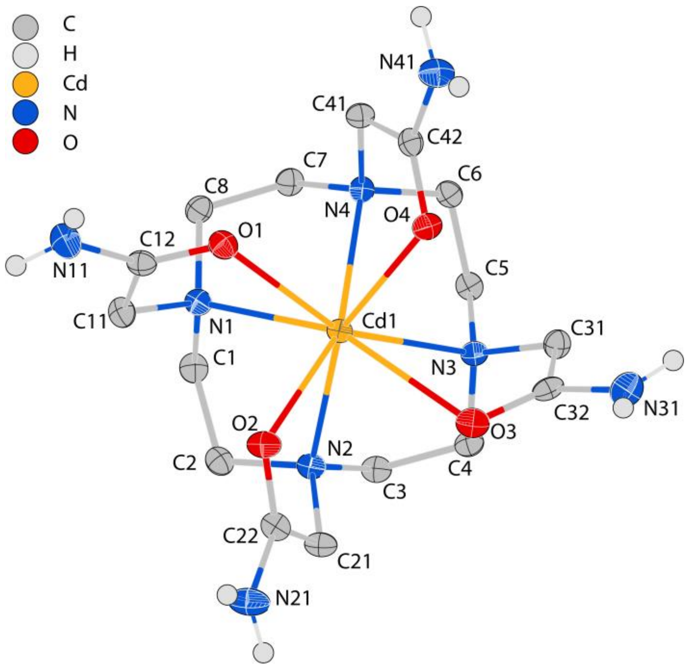

Figure S4. View of the complex [Cd(**dotam**)]$^{2+}$ cation in the structure of [Cd(**dotam**)][$CdCl_4$]·0.5H_2O with atom labelling necessary for the following discussion.

Table S4. Selected geometrical parameters in the structure of [Cd(**dotam**)][$CdCl_4$]·0.5H_2O.

Distances, Å			
Cd1–O1	2.5000(14)	Cd1–N1	2.4349(15)
Cd1–O2	2.3708(13)	Cd1–N2	2.4108(15)
Cd1–O3	2.5836(14)	Cd1–N3	2.4459(15)
Cd1–O4	2.3223(13)	Cd1–N4	2.4566(15)
Cd1–Q_O	1.322	Cd1–Q_N	1.246
Cd2–Cl1	2.489(7)	Cd2–Cl3	2.4421(6)
Cd2–Cl1B	2.49(2)	Cd2–Cl4	2.4467(7)
Cd2–Cl2	2.4400(5)		
Angles, °			
O1–Cd1–O3	117.96(4)	N1–Cd1–N3	118.33(5)
O2–Cd1–O4	110.72(5)	N2–Cd1–N4	118.69(5)
O1–O2–O3	95.82(5)	N1–N2–N3	90.05(6)
O2–O3–O4	82.51(5)	N2–N3–N4	89.93(6)
O3–O4–O1	97.99(5)	N3–N4–N1	90.05(6)
O4–O1–O2	83.67(5)	N4–N1–N2	89.96(6)
Cl1A–Cd2–Cl2	116.32(18)	Cl1B–Cd2–Cl4	104.9(7)
Cl1A–Cd2–Cl3	103.8(3)	Cl2–Cd2–Cl3	109.41(2)
Cl1A–Cd2–Cl4	109.5(3)	Cl2–Cd2–Cl4	104.74(2)
Cl1B–Cd2–Cl2	116.7(5)	Cl3–Cd2–Cl4	113.34(2)
Cl1B–Cd2–Cl3	107.9(8)		
Torsion angles, °			
N1–Q_N–Q_O–O1 [a]	−25.45	N3–Q_N–Q_O–O3 [a]	−24.49
N2–Q_N–Q_O–O2 [a]	−26.81	N4–Q_N–Q_O–O4 [a]	−26.19

[a] Q_N and Q_O are the centroids of N_4 and O_4 planes, respectively.

Table S5. Hydrogen bonds in the structure of [Cd(**dotam**)][$CdCl_4$]·0.5H_2O.

D–H···A	D–H	H···A	D···A	∠D-H-A
O1W–H1X···Cl2^{i}	1.13(7)	2.23(7)	3.337(4)	166(5)
O1W–H1Y···Cl1A^{ii}	1.14(8)	2.15(8)	3.172(13)	148(5)
O1W–H1Y···Cl1B^{ii}	1.14(8)	2.00(8)	3.00(4)	143(5)
N11–H11C···Cl4^{ii}	0.80(3)	2.58(3)	3.2731(18)	146(2)
N11–H11D···Cl1A^{iii}	0.85(3)	2.53(3)	3.369(7)	170(3)
N11–H11D···Cl1B^{iii}	0.85(3)	2.57(4)	3.41(2)	169(3)
N21–H21C···O2^{iii}	0.84(3)	2.09(3)	2.924(2)	171(3)
N21–H21D···Cl1A	0.80(3)	2.70(3)	3.470(12)	164(3)
N31–H31C···Cl1A^{iv}	0.824(15)	2.624(17)	3.429(7)	165.6(7)
N31–H31C···Cl1B^{iv}	0.824(15)	2.59(3)	3.40(2)	170.4(11)
N41–H41C···O3^{v}	0.91(2)	2.51(2)	3.040(2)	117.3(18)
N41–H41C···O4^{v}	0.91(2)	2.22(2)	3.075(2)	155.8(18)
N41–H41D···Cl4^{vi}	0.85(2)	2.54(2)	3.3831(19)	173(2)

Symmetry codes: i: $1 - x$, $1 - y$, $-z$; ii: $3/2 - x$, $-1/2 + y$, $1/2 - z$; iii: $1 - x$, $1 - y$, $1 - z$; iv: $-1 + x$, y, z; v: –x, $1 - y$, $1 - z$; vi: $1/2 - x$, $-1/2 + y$, $1/2 - z$.

Solution NMR spectroscopy

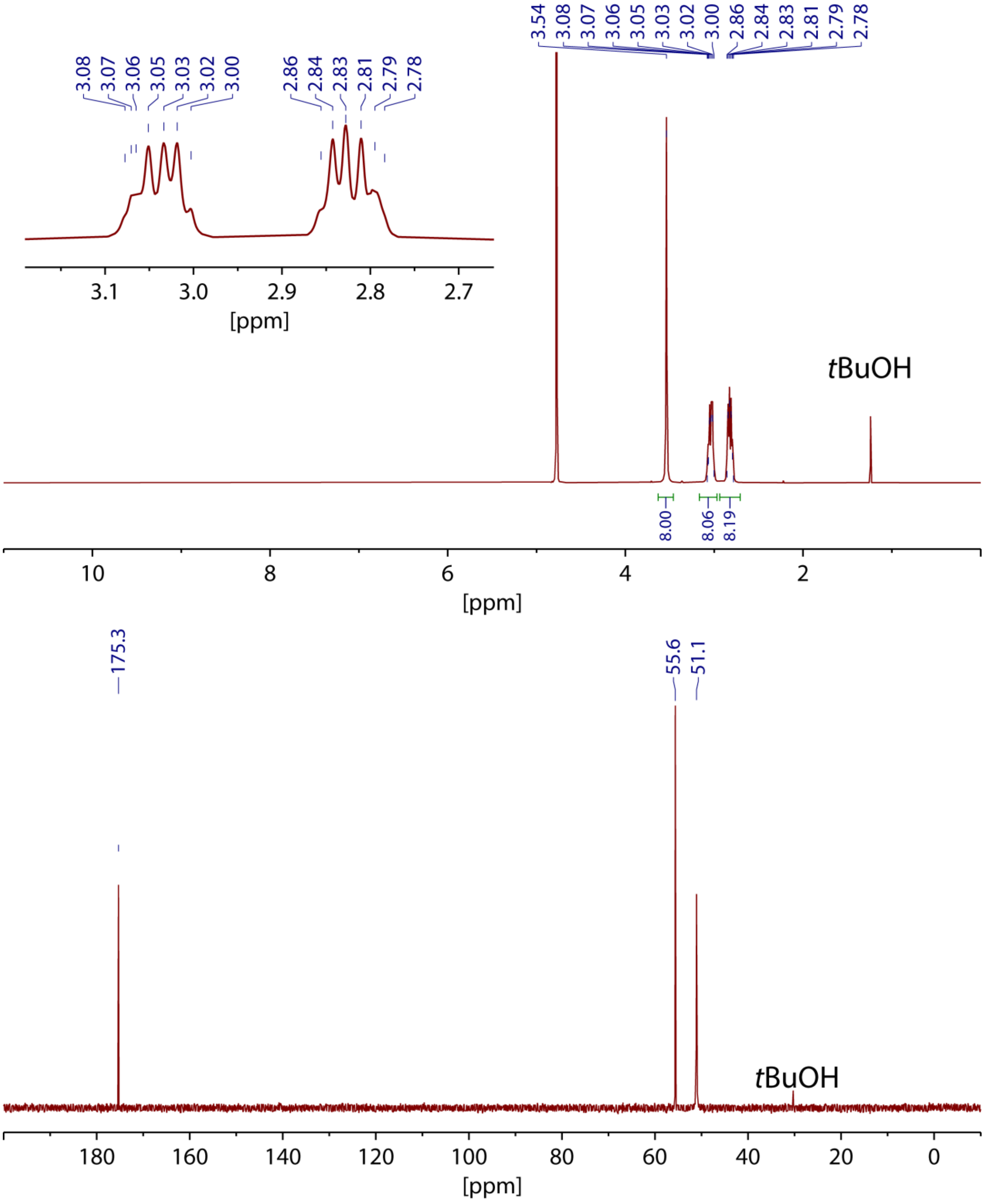

Figure S5. The ^{1}H (top) and ^{13}C{^{1}H} ((bottom) NMR spectra of the [Zn(**dotam**)][$ZnCl_4$] in solution (400 MHz, D_2O, 20 °C). The inset in the ^{1}H NMR spectrum shows the peaks assigned to the ring CH_2 protons.

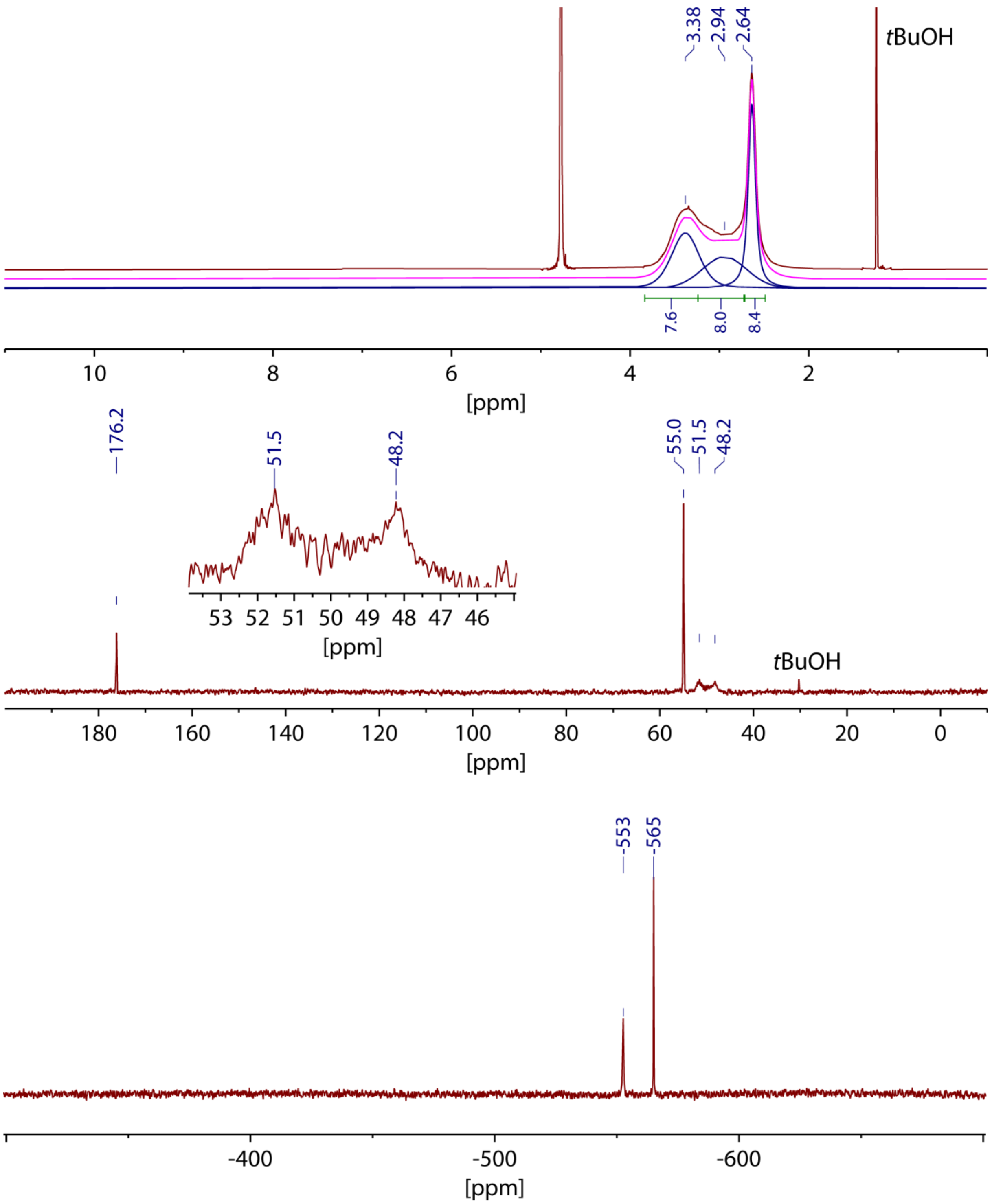

Figure S6. ^{1}H (top) (400 MHz, D_2O, 20 °C), $^{13}C\{^1H\}$ (middle) (101 MHz, D_2O, 20 °C) and ^{113}Cd (bottom) (133 MHz, D_2O, 20 °C) NMR spectra of the [Cd(**dotam**)][$CdCl_4$] in solution. The ^{1}H NMR spectrum was deconvoluted to obtain precise peak positions. Color code: **maroon** – experimental spectrum, **blue** – individual fitted peaks, **magenta** – sum of the fitted peaks. The inset in $^{13}C\{^1H\}$ NMR spectrum shows the peaks assigned to ring CH_2 carbons.

Solid-state NMR spectroscopy

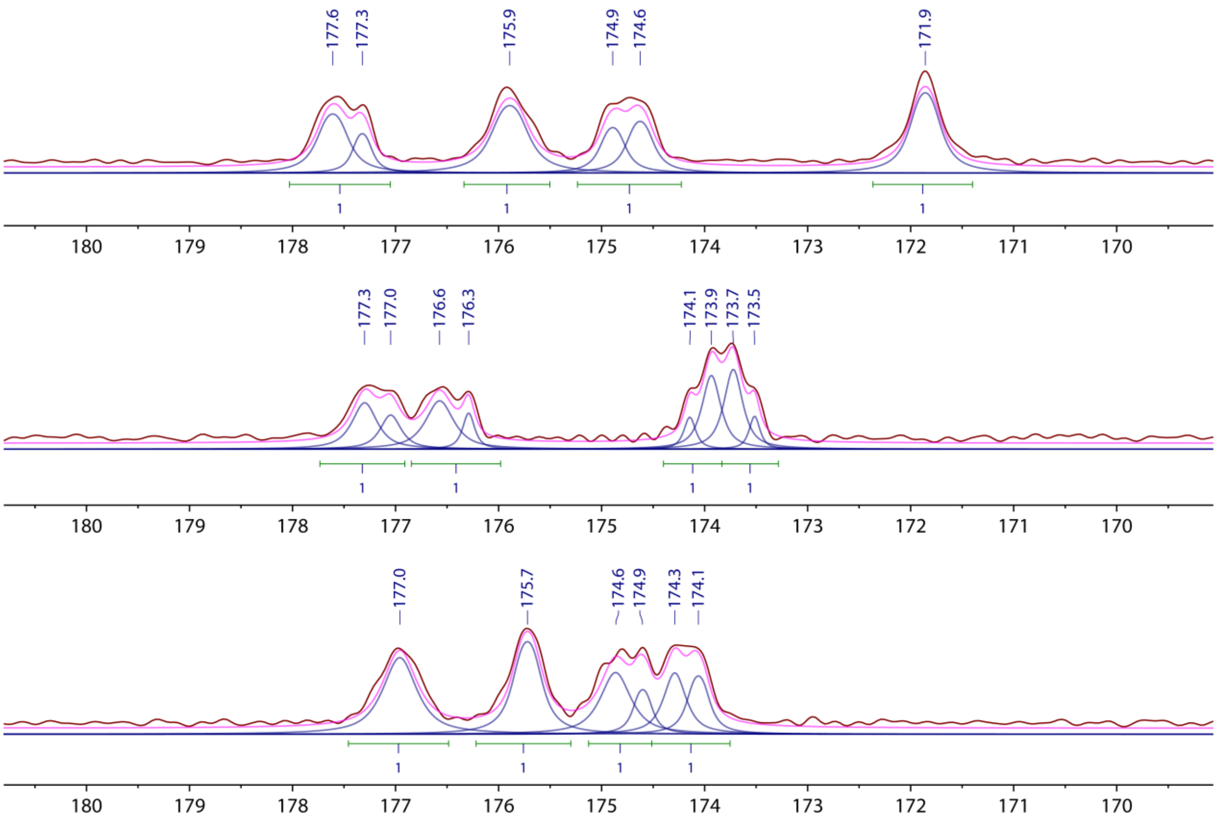

Figure S7. Expansion of the carbonyl region of ^{13}C CP/MAS NMR spectra (126 MHz, 10 kHz) of [Zn(**dotam**)][$ZnCl_4$]·2.5H_2O (top), [Cd(**dotam**)][$CdCl_4$]·0.5H_2O (middle) and [Hg(**dotam**)][Hg_3Cl_8]·3H_2O (bottom). color code: **maroon**: experimental spectra, **blue**: deconvoluted resonances of individual sites, **magenta**: sum of the deconvoluted resonances.

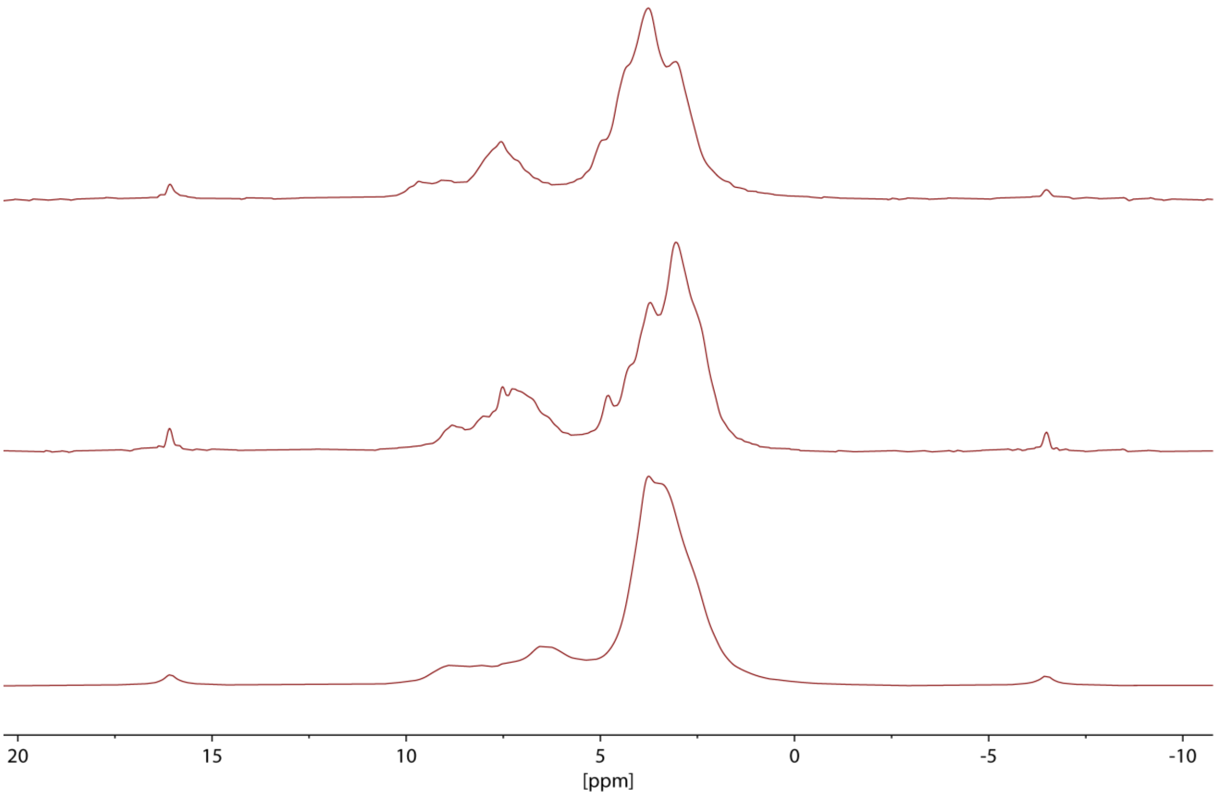

Figure S8. The ^{1}H DUMBO NMR spectra (700 MHz, 10 kHz) of [Zn(**dotam**)][$ZnCl_4$]·2.5H_2O (top), [Cd(**dotam**)][$CdCl_4$]·0.5H_2O (middle) and [Hg(**dotam**)][Hg_3Cl_8]·3H_2O.

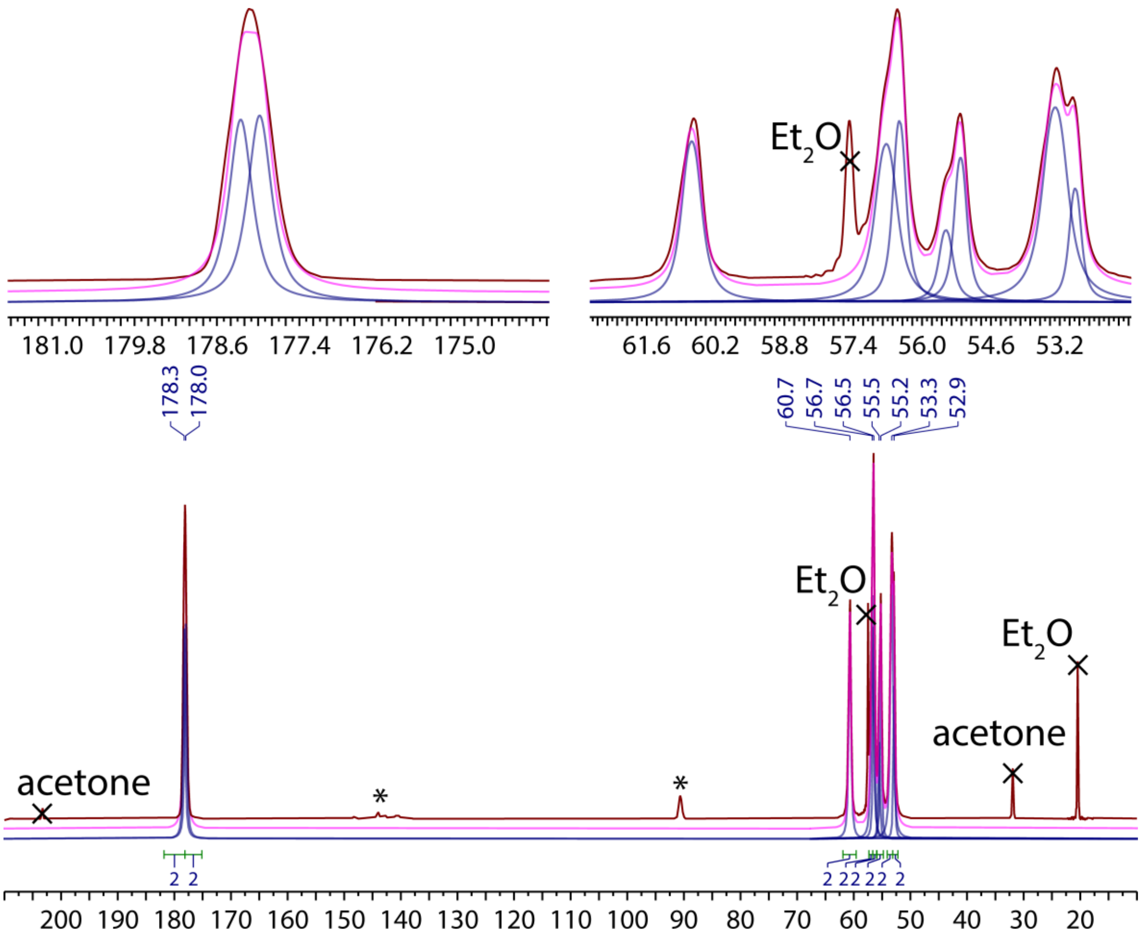

Figure S9. ^{13}C CP/MAS NMR spectrum (126 MHz, 10 kHz) of **dotam**. Color code: **maroon**: experimental spectra, **blue**: deconvoluted resonances of individual sites, **magenta**: sum of the deconvoluted resonances. The residual solvent resonances are marked with cross and spinning sidebands are marked with asterisks. The top charts show expansions of carbonyl (left) and methylene (right) regions.

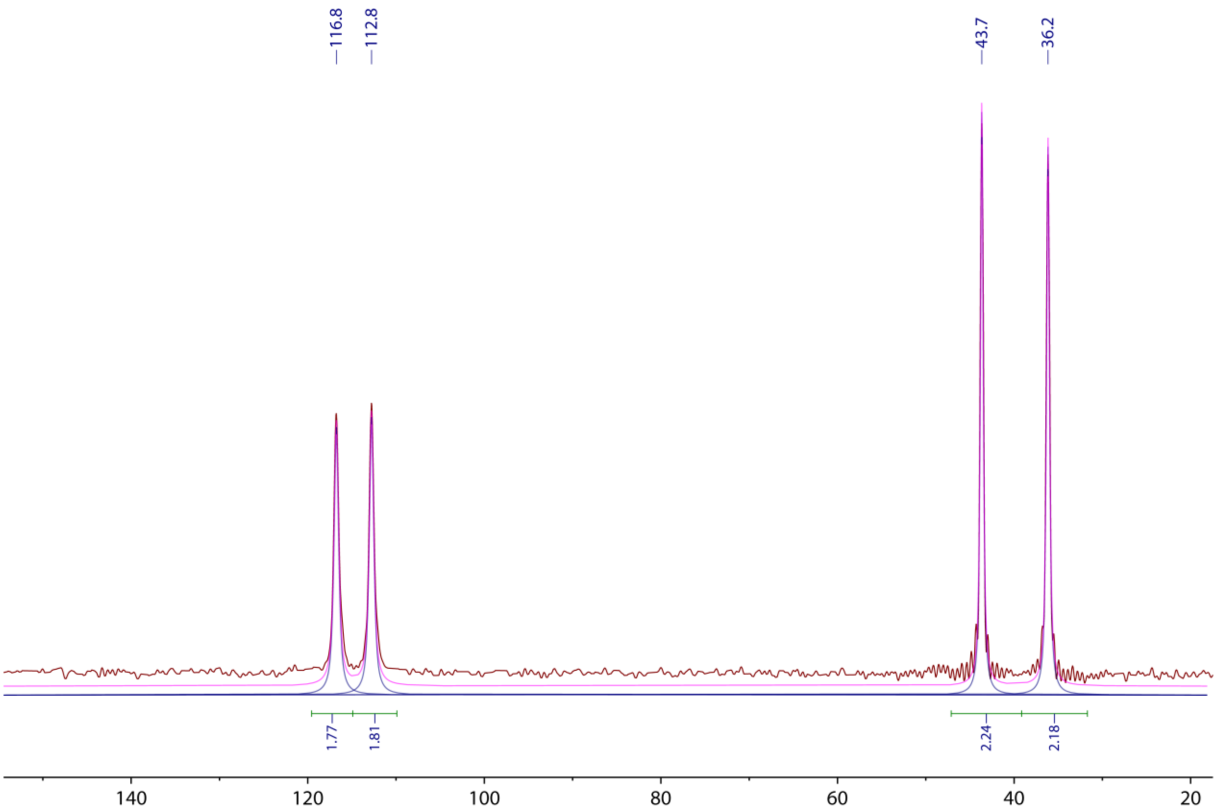

Figure S10. The ^{15}N CP/MAS NMR spectrum (51 MHz, 10 kHz) of **dotam**. Color code: maroon: experimental spectra, blue: deconvoluted resonances of individual sites, magenta: sum of the deconvoluted resonances.

Table S6. Fitting results of ^{13}C ss-NMR spectrum of [Zn(**dotam**)][$ZnCl_4$]·2.5H_2O. Site assignment was done according to the Castep calculations and is consistent with atom labels in **Figure S2**.

Carbonyl region								
#	Atom	Shift (ppm)	Height	Width	L/G	Area	A number of nuclei	Deviation
1.1	C22	177.60	425.1	42.4	0.8	11024	1	0.04
1.2		177.31	289.5	22.4	0.8	3965		
2	C12	175.89	481.4	47.6	0.8	14015	1	−0.02
3.1	C42	174.90	317.3	31.9	0.8	6178	1	0.02
3.2		174.63	377.4	36.5	0.8	8415		
4	C32	171.86	592.2	38.4	0.8	13887	1	−0.03
CH_2 region								
#	Atom	Shift (ppm)	Height	Width	L/G	Area	A number of nuclei	Deviation
1	C31	54.94	764.2	45.9	0.8	21459	1	0.32
2	C41	54.56	555.6	41.0	0.8	13926	1	−0.14
3	C21	54.11	672.3	42.3	0.8	17372	1	0.07
4	C11	53.75	541.1	30.7	0.8	10165	1	−0.37
5	C1, C6	52.79	1400.8	41.5	0.8	35558	2	0.10
6	C8	52.29	839.3	38.0	0.8	19491	1	0.20
7	C4	51.99	706.1	25.7	0.8	11099	1	−0.32
8	C2	51.11	728.6	37.2	0.8	16578	1	0.02
9	C5	49.94	706.8	37.5	0.8	16185	1	0.00
10	C7	48.91	646.0	40.4	0.8	15935	1	−0.02
11	C3	47.85	693.6	39.9	0.8	16902	1	0.04
Sum/RMSD							16	0.17

Table S7. Fitting results of ^{15}N ss-NMR spectrum of [Zn(**dotam**)][$ZnCl_4$]·2.5H_2O. Site assignment was done according to the Castep calculations and is consistent with atom labels in **Figure S2**.

Amide region								
#	Atom	Shift (ppm)	Height	Width	L/G	Area	A number of nuclei	Deviation
1	N11	116.08	522.0	15.8	0.8	6250	1	0.08
2	N21	111.13	487.9	15.1	0.8	5579	1	−0.04
3	N41B, N31B	108.99	530.0	28.4	0.8	11407	2	−0.02
Amine region								
#	Atom	Shift (ppm)	Height	Width	L/G	Area	A number of nuclei	Deviation
1	N1, N3	31.12	1959.9	12.7	0.8	18799	2	0.03
2	N2	28.99	955.1	11.7	0.8	8503	1	−0.07
3	N4	28.24	925.2	13.1	0.8	9156	1	0.00
Sum/RMSD							8	0.05

Table S8. Fitting results of ^{13}C ss-NMR spectrum of [Cd(**dotam**)][$CdCl_4$]·0.5H_2O. Site assignment was done according to the Castep calculations and is consistent with atom labels in **Figure S4**.

Carbonyl region								
#	Atom	Shift (ppm)	Height	Width	L/G	Area	A number of nuclei	Deviation
1.1	C22	177.30	90.8	38.2	0.8	2120	1	0.03
1.2		177.05	67.4	31.4	0.8	1293		
2.1	C42	176.57	94.5	43.3	0.8	2501	1	−0.01
2.2		176.29	71.1	18.5	0.8	802		
3.1	C32	174.14	65.7	18.8	0.8	754	1	−0.03
3.2		173.93	145.4	27.8	0.8	2468		
4.1	C12	173.72	156.6	27.6	0.8	2637	1	0.01
4.2		173.52	67.9	17.0	0.8	707		
CH_2 region								
#	Atom	Shift (ppm)	Height	Width	L/G	Area	A number of nuclei	Deviation
1	C41, C11	56.20	288.4	51.0	0.8	9411	2	0.19
2	C31	55.53	147.2	31.2	0.8	2807	1	−0.29
3	C21	54.92	168.4	38.4	0.8	3952	1	0.00
4	C4, C6	54.30	315.3	38.4	0.8	7753	2	−0.02
5	C2, C8	51.07	236.0	56.5	0.8	8148	2	0.03
6	C7	49.18	155.6	40.0	0.8	3801	1	−0.04
7	C5, C1	48.32	321.1	38.4	0.8	7888	2	0.00
8	C3	46.93	153.9	38.8	0.8	3644	1	−0.08
Sum/RMSD							16	0.10

Table S9. Fitting results of ^{15}N ss-NMR spectrum of [Cd(**dotam**)][$CdCl_4$]·0.5H_2O. Site assignment was done according to the Castep calculations and is consistent with atom labels in **Figure S4**.

#	Atom	Shift (ppm)	Height	Width	L/G	Area	A number of nuclei	Deviation
Amide region								
1	N11, N21	114.39	180.0	19.4	0.8	5304	2	0.05
2	N41	113.78	93.2	15.2	0.8	2147	1	−0.15
3	N31	109.50	108.7	16.0	0.8	2645	1	0.05
Amine region								
#	Atom	Shift (ppm)	Height	Width	L/G	Area	A number of nuclei	Deviation
1	N2	29.08	143.5	14.0	0.8	3044	1	0.23
2	N3	28.38	128.4	12.1	0.8	2362	1	−0.05
3	N4, N1	27.84	185.8	16.0	0.8	4508	2	−0.09
		Sum/RMSD					8	0.12

Table S10. DFT calculated ^{13}C chemical shieldings of [Zn(**dotam**)][$ZnCl_4$]·2.5H_2O. Calibration equation $\sigma = -1.00\ \delta + 172.85$ provided RMSD of 0.38 ppm.

Nucleus	GIPAW-PBE (ppm)	Experimental shift (ppm)
C22	−5.1597	177.53
C12	−3.4864	175.89
C42	−3.4148	174.74
C32	1.2636	171.86
C31	117.7376	54.94
C41	118.3065	54.56
C21	118.7156	54.11
C11	119.3732	53.75
C1	119.6820	52.79
C6	119.8049	52.79
C8	120.0611	52.29
C4	120.9079	51.99
C2	121.1140	51.11
C5	122.8412	49.94
C7	123.6500	48.91
C3	124.9880	47.85

Table S11. DFT calculated ^{15}N chemical shieldings of [Zn(**dotam**)][$ZnCl_4$]·2.5H_2O. Calibration equation $\sigma = -1.08\ \delta + 230.03$ provided RMSD of 1.81 ppm.

Nucleus	GIPAW-PBE (ppm)	Experimental shift (ppm)
N11	107.2497	116.08
N21	108.3415	111.13
N41B	108.9917	108.99
N31B	114.2011	108.99
N1	194.2803	31.12
N3	196.7197	31.12
N2	199.6839	28.99
N4	200.4473	28.24

Table S12. DFT calculated ^{13}C chemical shieldings of [Cd(**dotam**)][$CdCl_4$]·0.5H_2O. Calibration equation $\sigma = -1.01\ \delta + 172.8$ provided RMSD of 0.55 ppm.

Nucleus	GIPAW-PBE (ppm)	Experimental shift (ppm)
C22	−5.6164	177.21
C42	−4.9415	176.51
C32	−2.3658	173.98
C12	−2.1175	173.68
C41	116.3761	56.20
C11	116.7339	56.20
C31	116.8128	55.53
C21	118.1541	54.92
C4	118.6315	54.30
C6	118.9015	54.30
C2	119.6842	51.07
C8	121.4172	51.07
C7	123.3820	49.18
C5	123.5821	48.32
C1	124.2343	48.32
C3	125.1743	46.93

Table S13. DFT calculated ^{15}N chemical shieldings of [Cd(**dotam**)][$CdCl_4$]·0.5H_2O. Calibration equation $\sigma = -1.09\ \delta + 233.51$ provided RMSD of 0.56 ppm.

Nucleus	GIPAW-PBE (ppm)	Experimental shift (ppm)
N11	107.5018	114.39
N21	108.7163	114.39
N41	109.1461	113.78
N31	114.9545	109.50
N2	201.7025	29.08
N3	201.8393	28.38
N4	203.2802	27.84
N1	203.5580	27.84

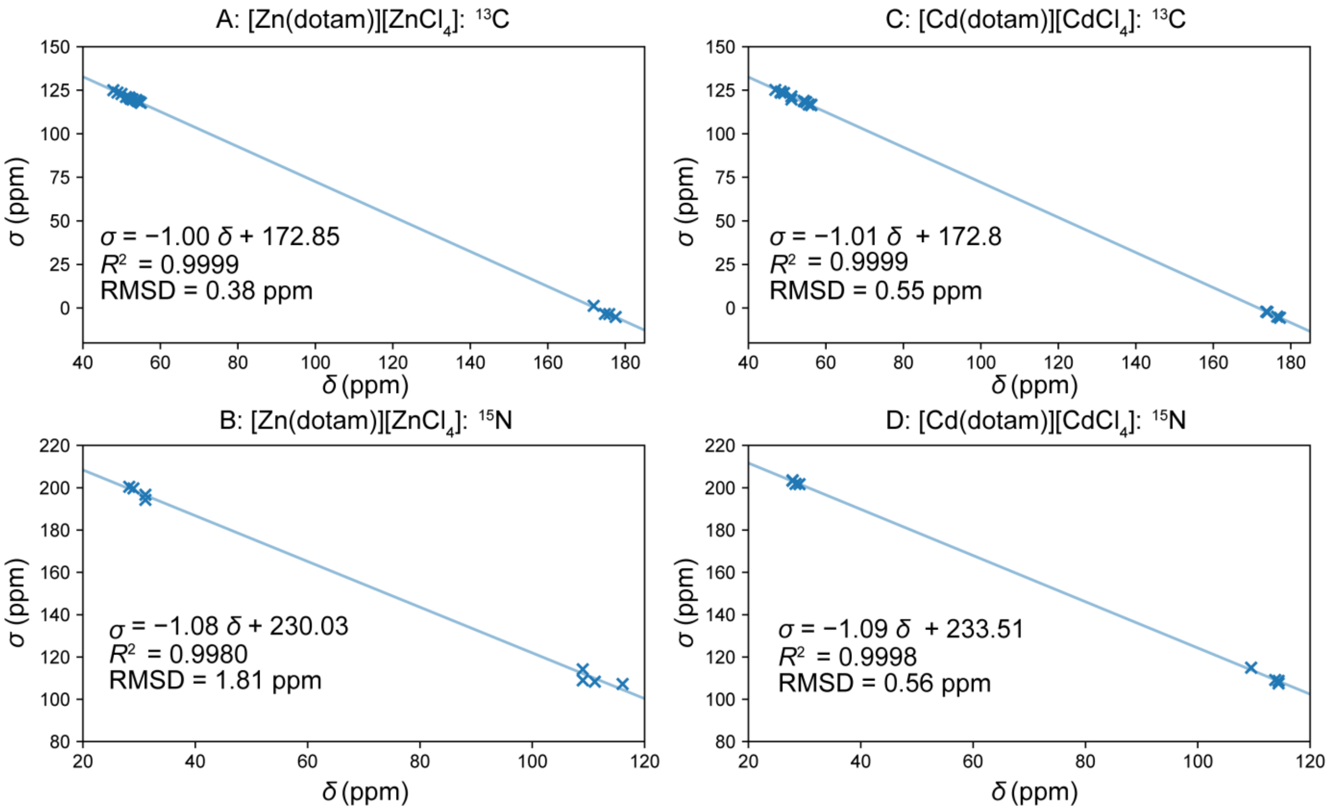

Figure S11. Plots of ^{13}C and ^{15}N experimental shift *vs.* calculated shielding for the studied compounds, demonstrating a good match between the experimental spectra and XRD structures.
